# Neurotrophin signaling is a central mechanism of salivary dysfunction after irradiation that disrupts myoepithelial cells

**DOI:** 10.1038/s41536-023-00290-7

**Published:** 2023-03-25

**Authors:** Alejandro M. Chibly, Vaishali N. Patel, Marit H. Aure, Mary C. Pasquale, NIDCD/NIDCR Genomics and Computational Biology Core, Robert J. Morell, Daniel Martin Izquierdo, Erich Boger, Gemma E. Martin, Mousa Ghannam, Julianne Andrade, Noah G. Denegre, Colleen Simpson, David P. Goldstein, Fei-Fei Liu, Isabelle M. A. Lombaert, Matthew P. Hoffman

**Affiliations:** 1 Matrix and Morphogenesis Section, National Institute of Dental and Craniofacial Research, National Institutes of Health, Bethesda, MD 20892 USA; 2 Department of Otolaryngology-Head & Neck Surgery, Princess Margaret Cancer Center, Toronto, ON M5G 2C4 Canada; 3 Department of Radiation Oncology, Princess Margaret Cancer Center, Toronto, ON M5G 2M9 Canada; 4 Department of Biologic and Material Sciences, School of Dentistry, University of Michigan, Ann Arbor, MI 48109 USA; 5 Biointerfaces Institute, University of Michigan, Ann Arbor, MI 48109 USA; 6 Genomics and Computational Biology Core, National Institute on Deafness and Other Communication Disorders, National Institutes of Health, 35A Convent Drive, Room 1F-103, Bethesda, MD 20892-3729 USA; 7 Genomics and Computational Biology Core, National Institute on Dental and Craniofacial Research, National Institutes of Health, Bethesda, MD 20892 USA

**Keywords:** Stress signalling, Transcriptomics

## Abstract

The mechanisms that prevent regeneration of irradiated (IR) salivary glands remain elusive. Bulk RNAseq of IR versus non-IR human salivary glands showed that neurotrophin signaling is highly disrupted post-radiation. Neurotrophin receptors (NTRs) were significantly upregulated in myoepithelial cells (MECs) post-IR, and single cell RNAseq revealed that MECs pericytes, and duct cells are the main sources of neurotrophin ligands. Using two ex vivo models, we show that nerve growth factor (NGF) induces expression of MEC genes during development, and upregulation of NTRs in adult MECs is associated with stress-induced plasticity and morphological abnormalities in IR human glands. As MECs are epithelial progenitors after gland damage and are required for proper acinar cell contraction and secretion, we propose that MEC-specific upregulation of NTRs post-IR disrupts MEC differentiation and potentially impedes the ability of the gland to regenerate.

## Introduction

Chronic IR damage to the salivary glands (SG) as a side effect of head and neck cancer therapy results in hyposalivation due to a major depletion of secretory acinar cells, which fail to regenerate. The description of IR damage to SG focuses on this obvious acinar cell loss and the excess deposition of extracellular matrix resulting in fibrosis, but IR also disrupts gland innervation, increases vascular permeability causing interstitial edema and marked immune infiltration^1–3^. While some level of acinar cell proliferation and (trans)-differentiation has been observed post-IR, it is still unclear why the gland is unable to fully repair itself. It is not known why human acinar cells do not regenerate after IR damage while myoepithelial cells (MEC) and ducts are maintained. Therefore, analyzing the global transcriptional landscape in response to IR damage in humans is an important first step to identify potential epithelial signaling pathways that are disrupted and result in the lack of regeneration.

Lineage tracing studies in mice have shown that Mist1+ acinar cells undergo self-duplication and KRT5/14+ cells (i.e., representing ductal cells and MECs) replenish their own compartment during homeostasis as well as in models of reversible SG injury (e.g., duct ligation) without damage to the blood supply or nerves^4–6^. In contrast, newly formed mitotically active acinar cells can derive from both Mist1+ acinar and KRT5/14+ cells after a single 15 Gy dose of IR damage to murine submandibular glands (SMGs)^4^ and in severe duct ligation models that compromise the blood supply and nerves. Interestingly, ~49% of the regenerated acini derived from MECs in the severe duct ligation model, either through direct acinar conversion (~5%) or via indirect conversion to a ductal/acinar bipotent progenitor (91%)^7^. The latter observation is analogous to MECs in the submucosal gland, which give rise to themselves during homeostasis but can regenerate the airway upon injury through conversion into another epithelial cell type^8^. These findings suggest that plasticity of multiple cell types, and MECs in particular, contribute to the regeneration of acinar cells^7,9^. However, given that endogenous acinar regeneration is minimal in SMGs after severe injury conditions, it is plausible that mechanisms of acinar cell self-renewal or MEC plasticity are affected.

In addition to cell-autonomous mechanisms that may prevent effective epithelial regeneration post-IR, the complex alterations to the gland microenvironment are likely to disrupt the signals that would otherwise instruct epithelial cells to undergo effective differentiation programs to allow the gland to regenerate. For instance, we previously demonstrated that both mesenchymal and neuronal-mediated signaling pathways are essential for the differentiation of various epithelial progenitors^10,11^. Specifically for salivary glands, the nerve growth factor (NGF) and glial-derived neurotrophic factor (GDNF) family that includes Neurturin (NRTN) play important roles in healing after injury.

NRTN and its receptor, GFRa2, play an important role in epithelial regeneration in ex vivo models of gland damage^10^. NRTN protects the gland from IR damage and facilitates regeneration ex vivo by stimulating parasympathetic innervation, which in turn is required for progenitor cell maintenance and expansion^10,11^. In addition, NRTN can be used for gene therapy to restore salivary flow after IR in both mice and minipigs^12,13^. The SG is also a rich source of other neurotrophins such as nerve growth factor (NGF), which was originally isolated from snake venom glands and murine SMGs^14^ and plays a role in oral wound healing^15^. Recently NGF was shown to protect salivary gland cell lines and murine SGs from apoptosis after acute IR damage^16^. These preclinical observations highlight the importance of niche signals such as neurotrophic factors in the context of SG function and regeneration, but how the neurotrophin pathway is affected in clinical settings, such as irradiated human salivary glands, and how their alterations potentially influence cell plasticity is unclear.

The demonstrated plasticity of MECs and duct cells to form acinar cells in preclinical injury models suggests that the SG has an innate ability to regenerate. However, given that severely IR human SGs do not regenerate, a major unanswered question of clinical relevance is to define what cellular plasticity and associated differentiation programs are disrupted post-IR. To address this question, we performed bulk RNAseq in control (i.e., non-IR) and IR human parotid gland (PG) and SMG and used bioinformatic approaches to define the signaling pathways affected by IR. We leverage this information with the single cell (sc) RNAseq atlas of murine SMG development^17^ and the Tabula Sapiens^18^ to predict the cellular localization and potential communication networks mediated by the differentially expressed genes. Our analyses led us to investigate the role of upregulated neurotrophin signaling in MECs through a combination of ex vivo organ cultures and isolated primary adult MEC cultures. We propose a working model in which upregulation of NGF and neurotrophin signaling is linked to an alteration in MEC plasticity post-IR, which may impede endogenous gland regeneration.

## Results

### Neurotrophin signaling is a central pathway disrupted by IR

**Fig. 1:**
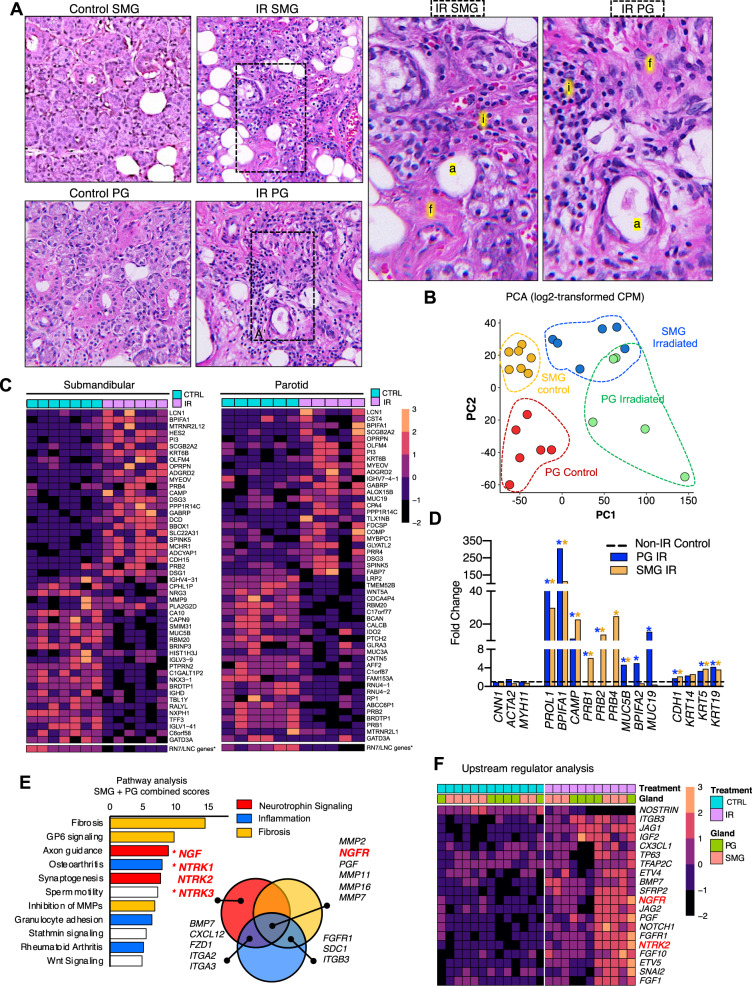
Neurotrophin signaling is a key pathway dysregulated post-IR. **A** Representative H&E staining of control and IR human SMG and PG. Areas in dotted boxes of IR SMG and IR PG are enlarged in right two panels to highlight increased adiposity (a), immune infiltration (i), and fibrosis (f). **B** PCA plot of log-transformed CPM counts from bulk RNAseq analysis of human biopsies. **C** Heatmaps of the top 50 DEGs by fold change in irradiated glands compared to controls. The color scale represents scaled gene expression values. *Long non-coding RNAs, small nuclear RNAs, SNORAs, and other pseudogenes were overrepresented among downregulated genes and were removed from heatmap (see Supplementary Data 1 for complete list); a representative expression profile is shown at the bottom of the heatmaps. **D** Bar graph with fold change gene expression of selected genes in irradiated glands (*n* = 6 IR-SMG; *n* = 5 IR-PG) compared to controls from RNAseq analysis. Stars denote statistical significance (adjusted *p* < 0.05, statistical analysis with EdgeR-DeSeq2 (Wald test p-value and Benjamini-Hochberg adjusted *p*-value)). **E** Results from Ingenuity Pathway Analysis (IPA) Software showing common pathways dysregulated in PG and SMG. A combined score was calculated by adding the –Log(*p*-val) for a given pathway in both glands. The Venn diagram highlights the overlap of genes associated with the top dysregulated pathways. * NGF and NTRK1 were only significant in IR-SMG and NTRK3 in IR-PG. **F** Heatmap showing results from Upstream Regulator analysis with IPA. Only genes that met the criteria for significance in our dataset (*p*-value < 0.05 and fold change > 2) and that were predicted to be upstream of DEGs in both glands are shown. Analysis was adjusted to only predict genes annotated to function as growth factors, receptors, and transcription factors, based on IPA’s database.

To unbiasedly identify dysregulated signaling pathways in chronic IR-induced salivary glands, biopsies of control and irradiated PG and SMG were collected 4 months to 7 years post-IR from patients undergoing surgery for head and neck cancer and analyzed by RNAseq (Fig. 1). All IR PGs and SMGs stained with hematoxylin and eosin showed the expected alterations in gland architecture, including reduced acinar cells, increased fibrosis, adiposity and immune infiltration, as well as blood vessel abnormalities, consistent with previous clinical observations (Fig. 1A). The reduced acinar cell density after IR was also apparent by E-cadherin immunostaining, while epithelial ducts and stromal tissue remained the predominant feature of the residual tissue (Supplementary Fig. 1A). Clinical characteristics from each biopsy are described in Supplementary Fig. 1B. We also performed the well-established sphere culture method on primary cells dissociated from selected biopsies to evaluate the level of epithelial stem/progenitors^19,20^, which confirmed the overall reduced number of epithelial stem/progenitor cells from irradiated glands compared to controls (Supplementary Fig. 1C–E).

Only biopsies with an RNA integrity number >7 were considered for RNAseq analysis. These included 13 SMG (7 control + 6 IR) and 11 PG (6 control + 5 IR) samples (Supplementary Fig. 1B). Most samples obtained were from male subjects due to the higher prevalence of head and neck cancer in the male sex, but this did not influence the data as no sex-dependent clustering of samples by principal component analysis (PCA) was observed (Supplementary Fig. 2A). PCA analysis did separate samples based on both treatment and type of gland (Fig. 1B), while the time between the last dose of radiation and sample collection did not (Supplementary Fig. 2A). As a result, differential expression analysis was performed independently for each type of gland and by normalizing all IR samples against their respective controls.

We identified 1,643 DEGs in IR-PG and 1,471 DEGs in IR-SMG and heatmaps of the 25 most highly down and upregulated genes are shown (Fig. 1C, All data in Supplementary Data 1). The downregulation of many ribosomal *RN7* and long non-coding *LNC* pseudogenes was evident in both glands. Out of the top 30 downregulated genes in each gland, there were 17 *RN7S* pseudogenes in the IR SMG and 18 in the IR PG. Only one representative gene was included in DEG heatmaps and the rest are listed in Supplementary Data 1. Both IR glands showed upregulation of a common cluster of genes, including lipocalin-1 (*LCN1*), BPI Fold Containing Family A Member 1 (*BPIFA1*), peptidase inhibitor 3 (*PI3*), and secretoglobin family 2A member 2 (*SCGB2A2*), all of which are secreted salivary proteins. Other genes that were upregulated included *ASCL3*, a marker of salivary ionocytes and *LRG5*, which marks progenitor cells in the intestine^21–23^, as well as members of the frizzled family (*FRZB* and *SFRP2*) associated with mesenchymal WNT signaling (Supplementary Data 1).

The expression of MEC markers (*CNN1*, *ACTA2* and *MYH11*) were unchanged post-IR by qPCR, whereas ductal genes *KRT5* and *KRT19* were increased (Fig. 1D). Moreover, gland-specific dysregulation of serous and mucous secretory genes was evident. The mucin gene Opiorphin (*OPRPN*, also known as *PROL1* or mucin 10 (*Muc10*) in mouse) and cathelicidin antimicrobial peptide (CAMP) were among the top upregulated genes in both IR-PG and IR-SMG (Fig. 1C, D). Salivary proline-rich protein genes (*PRB1–4*), which are produced by acinar cells to regulate calcium concentration in saliva, were downregulated in IR-PG but upregulated in IR-SMG, and the mucin genes *MUC5B* and *MUC19* were downregulated in IR-SMG but upregulated in IR-PG (Fig. 1D). These gland-specific differences in the expression of secretory genes post-IR could reflect the distinct serous versus seromucous nature of the PG and SMG.

The analysis of DEGs highlights the multifactorial nature of the mechanisms that may be involved in chronic IR-induced salivary dysfunction. Using Ingenuity Pathway Analysis (IPA) software, we identified 102 signaling pathways overrepresented in the IR-PG DEGs and 65 pathways in IR-SMG DEGs (Supplementary Tables 1 and 2, Supplementary Data 2), and focused on the common IR-dysregulated pathways across both glands. Because of the redundancy of genes across multiple pathways, and the presence of interrelated pathways, we broadly grouped them into three major clusters of signaling processes corresponding to neurotrophic signaling (including Axon guidance, and Synaptogenesis), fibrosis (including the IPA annotation of Fibrosis, GP6 signaling and Inhibition of MMPs), and inflammation (including Osteoarthritis, Granulocyte Adhesion and Rheumatoid Arthritis) (Fig. 1E, Supplementary Fig. 2B). These pathways are consistent with the pathology of IR glands showing increased fibrotic tissue and immune infiltration, and with the known importance of innervation and neurotrophic factors in salivary gland function^10,12,24^. Eighty-eight genes were part of the top dysregulated pathways in both IR glands (Supplementary Fig. 2C), and a subset of 6 genes (*NGFR, PGF, MMP2, MMP7, MMP11, MMP16*) was common across the three major signaling clusters in both glands as visualized in the Venn Diagrams (Fig. 1E, Supplementary Fig. 2D–E), suggesting they could play a conserved central role post-IR. All other genes associated with each of the top three signaling clusters in both IR glands are visualized in the heatmap and chord plot in Supplementary Fig. 2E–F.

We applied IPA’s upstream analysis feature to identify genes that are likely to signal upstream of the IR-induced DEGs and pathways. Because signaling pathways are often triggered by the interaction between cell receptors with their respective ligands and culminate in the activation of transcription factors to regulate gene expression, we limited our prediction of upstream regulators to growth factors, receptors, and transcription factors (Supplementary Table 3, Supplementary Data 3). A heat map shows the 20 upstream regulators common to both glands, which include genes associated with neurotrophin signaling (*NGFR, NTRK2*), Fibroblast Growth Factor signaling (*FGFR1, FGF1*, *FGF10, ETV4, ETV5,* and *BMP7*), and Notch pathway (*NOTCH1, JAG1, JAG2*, and *SNAI2*) (Fig. 1F). Strikingly, NGF and all canonical neurotrophin receptors, herein referred to as NTRs including NGFR and NTRK1–3 (protein names, TRKA, TRKB, and TRKC), were identified either as upstream regulators (NGFR and NTRK2), in the 6-gene subset associated with all dysregulated pathways (NGFR), or in the neurotrophic signaling cluster (NTRK1, NTRK2, NTRK3, NGF) (Fig. 1E, F). Due to the prominent changes in NGF and NTRKs, we focused our further analysis on this signaling pathway.

### Neurotrophin receptors are upregulated in salivary MECs post-IR in humans

**Fig. 2:**
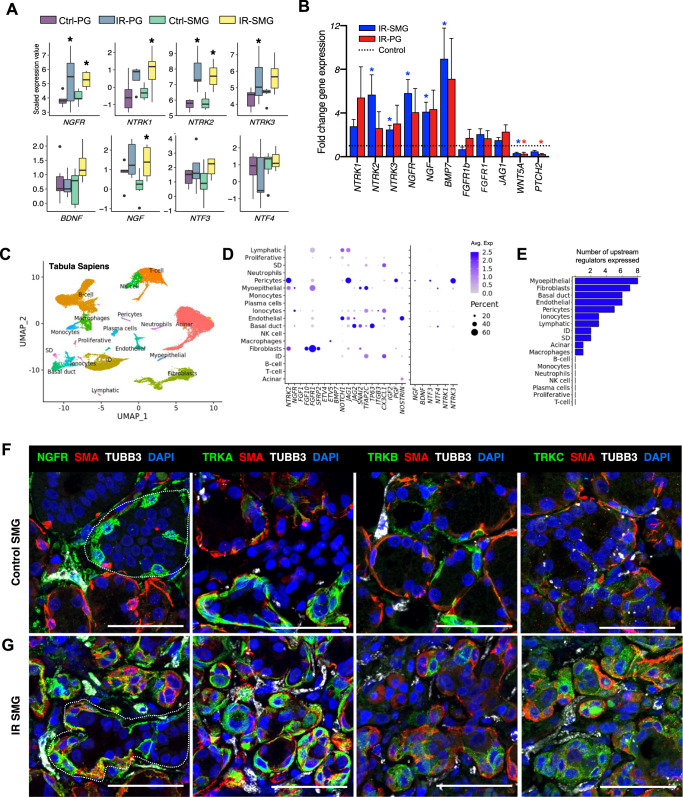
NTRs are upregulated in MECs post-IR. **A** Box plots showing the median expression of neurotrophin signaling genes in bulk-RNAseq data from human salivary glands (*n* = 7 SMG, *n* = 6 SMG-IR, *n* = 6 PG, *n* = 5 PG-IR). The box represents the interquartile range and the bars span the minimum and maximum values. Star denotes statistical significance (*p*-value < 0.05 and fold change > 2, EdgeR-DeSeq2-Limma pipeline). **B** qPCR analysis of selected genes in irradiated human SMG and PG samples (*n* = 4 per group). Gene expression was normalized to *GAPDH* and non-irradiated controls. Statistical significance (*p*-value < 0.05) was determined by two-tailed *t*-test with log-transformed fold changes and is shown with a star above bars. Error bars represent standard error of the mean (SEM). **C** UMAP of scRNAseq from human SG from the Tabula Sapiens. **D** Balloon plot of expression of IPA’s upstream regulator and neurotrophin signaling genes in Tabula Sapiens. Color scale represents average scaled expression and size reflects percentage of cells expressing a given gene. **E** Number of IPA’s upstream regulator genes expressed in each cell type. **F**, **G** Immunofluorescence staining for NGFR and neurotrophin receptors (green) in control (**A**) and IR (**B**) human SMG. Smooth muscle actin (SMA, red) labels myoepithelial cells, and nerve-specific tubulin beta 3 (TUBB3, white) labels peripheral nerves. Scale bar = 50 μm.

We first analyzed NTRK-relevant key players in the RNAseq data (Fig. 2A) and validated them by qPCR in an independent set of biopsies (Fig. 2B). Receptors NGFR, NTRK1, -2, and -3 were significantly upregulated in IR PG/SMG samples compared to their naïve counterpart, and their increased trends were confirmed by qPCR. Ligand NGF also significantly increased in both assays, while BDNF, NTF3 and NTF4 were not found to be significantly different. As a reference, other DEGs from the RNAseq analysis, including the significant upregulation of *BMP7* and two of the top downregulated genes post-IR in PGs, *WNT5A* and *PTCH2* (Fig. 1C), were also confirmed by qPCR. To further reveal whether NGF/NTRK changes were equally distributed across all IR samples, we divided IR biopsies into acute injured (i.e., collected ≤1-year post-injury) and chronically injured ones (>1 year post-IR) (Supplementary Fig. 3A). While the expression of NGF/NTRKs was significantly higher in acute IR samples as compared to naïve biopsies, it remained elevated during the chronic state (Supplementary Fig. 3A), suggesting that neurotrophin signaling remains chronically dysregulated after IR damage. As we observed that PG biopsies, which generally contained more fat and connective tissue, generated more fluctuation in gene expression across our various analysis as compared to SMGs, we primarily continued with SMG.

To gain a better understanding of the cell types involved with NTRK signaling, which is well known for its function in peripheral and central nerves^25,26^, we evaluated the expression of neurotrophin signaling genes and upstream regulators from our IPA analysis in the scRNAseq data from Tabula Sapiens human salivary glands^18^. One limitation of this dataset is that it did not contain any nerve or glial cells from salivary gland tissue, and many of the represented cell types were exclusively from either PG or SMG (Fig. 2C, Supplementary Fig. 3B). We looked at genes that were expressed in at least 15% of cells within a cluster and found that MECs expressed the most upstream regulator genes relative to all other cell types (Fig. 2D, E). More specifically, NGFR was differentially expressed in MECs and ionocytes, and NTRK2 in MECs, pericytes, and fibroblasts (Fig. 2D). Notch signaling genes were primarily expressed by endothelial cells and pericytes; FGF signaling genes were strongly expressed in various cell types but most notably in fibroblasts; and the remaining upstream regulator genes from IPA were expressed by MECs, fibroblasts, pericytes, endothelial cells, basal ducts, and ionocytes (Fig. 2D). Evaluation of other neurotrophin genes showed that NTRK3 was expressed by pericytes and MECs, whereas NTRK1 was not detected. Neurotrophin ligands NGF, BDNF, and NTF3/4 were also expressed in MECs, pericytes, and duct cells.

Next, we evaluated the NTR pathway on protein level in human SMGs. Immunofluorescent staining of control SMGs (Fig. 2F) confirmed expression of NGFR in duct cells and peripheral nerves (TUBB3+), although it was not detected in MECs. TRKA was found in nerves and MECs (SMA+), and both TRKB and TRKC in MECs (Fig. 2F, separated channels are shown in Supplementary Fig. 3). Surprisingly, after IR damage, NGFR and TRKA became highly upregulated in MECs (Fig. 2G, Supplementary Fig. 3C), while both TRKB and TRKC were upregulated in MECs and other epithelial cells (Fig. 2G, Supplementary Fig. 3C).

These unexpected findings combined highlight the complexity of neurotrophin signaling in salivary glands, where both paracrine and autocrine mechanisms involving multiple cell types are likely to take place. Furthermore, they show that upon IR injury, chronic upregulation of all four NTRs occurs in MECs and is associated with the loss of organ function post-IR. MECs are relatively understudied but it has been shown that their plasticity can contribute to acinar cell regeneration, making them an attractive population for further investigation. In addition, given that the functions of NTRs in MECs is unknown, our study aimed to investigate MECs’ response to neurotrophin signaling pathway manipulation. More specifically, we aimed to investigate how neurotrophin signaling manipulation would affect the development and plasticity of MECs in the absence of IR.

### The murine SMG model confirms that MECs are a major neurotrophin signaling hub

Due to the limited supply of human SG biopsies, we turned to the mouse model to address our questions on the function of neurotrophin signaling in naïve MECs. First, to determine whether the murine SMG is an appropriate experimental model to investigate the role of neurotrophin signaling in MECs and to investigate a potential role of this pathway in MEC development, we evaluated the expression of neurotrophin genes with published scRNAseq on embryonic and postnatal mouse SMG^17^.

**Fig. 3:**
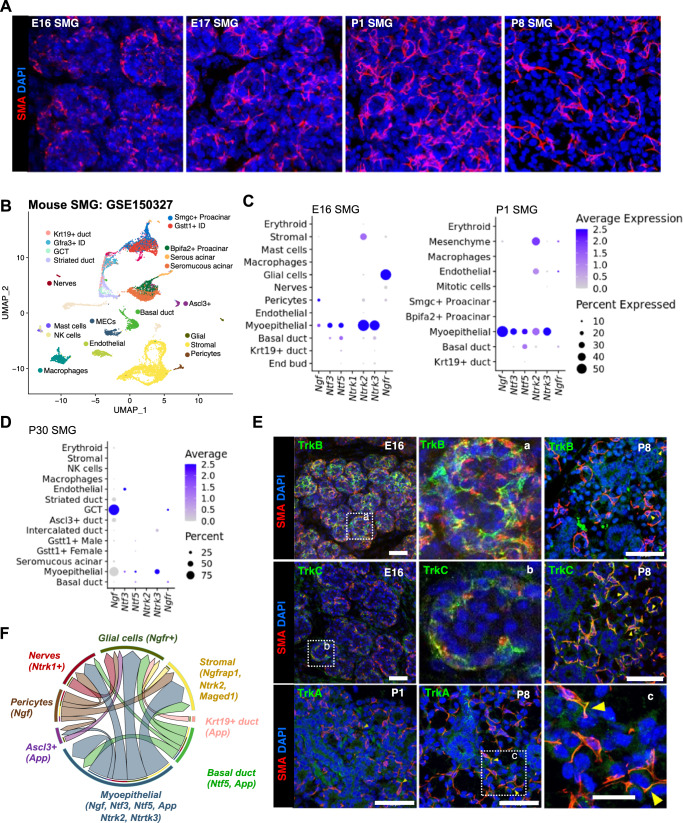
Murine SMG is an ideal model to investigate neurotrophin signaling in healthy salivary glands and confirms that MECs are a major neurotrophin signaling hub. **A** Immunostaining of SMA in mouse SMG at embryonic days E16 and E17, and postnatal days P1 and P8. **B** scRNAseq from mouse SMG (GSE150327). **C**, **D** Balloon plot of expression of neurotrophin signaling genes in mouse SMG at selected developmental stages. **E** Immunostaining for SMA (red), TrkA, TrkB and TrkC (green) and nuclei (DAPI, blue) in mouse SMG at E16, P1 and P8. White boxes (a-c) are shown enlarged. Scale bar, 50 µm except c) Scale bar, 20 µm. **F** Representation of putative ligand-receptor interactions between MECs and other cell types via neurotrophin signaling genes. Arrows point from the source of ligands in the direction of the receptor and the thickness of the arrow is relative to the number of potential interactions between two cells. Analysis and plots generated with the LigandReceptor script available on GitHub (https://github.com/chiblyaa/LigandReceptor). A list of curated pairs was obtained from Ramilowski et al.^50^.

As previously described, SMA immunostaining during mouse SMG development indicated that MECs appear in the outer layer of the endbud around E16^27^ and progress to form stellate cells that wrap around the developing acinar structures from E17 to postnatal days P1-P8 (Fig. 3A). Thus, we focused on the mouse scRNAseq data from embryonic day 16 (E16) and postnatal SMG (Fig. 3B). Expression of neurotrophin genes in murine SMG (Fig. 3C, D) was overall consistent with our findings in the Tabula Sapiens. One major difference was that we could also see the expression of *Ngfr* in nerves and glial cells, which was not observed in the human dataset as those cells were not captured in that set-up (Fig. 3C). The most obvious difference between mouse and human data was noted in pericytes, which did not show strong expression of neurotrophin genes in the mouse model. Instead, MECs had the highest relative expression of *Ntf3, Ntf5, Ntrk2*, and *Ntrk3* (Fig. 3C, D) amongst all captured human cell types. It is worth noting that in addition to MECs, granular convoluted ducts (GCT) had the highest expression of *Ngf* in P30 mouse SMGs (Fig. 3D); however, this cell type is not present in human glands.

We next confirmed gene expression analysis on protein levels. Co-expression of TrkB and TrkC (products of *Ntrk2* and *Ntrk3*, respectively) in SMA + MECs was confirmed in E16 mouse SMG via immunofluorescent staining (Fig. 3E). TrkA was not detected in E16 mouse SMG (not shown) but it was detected at low levels in some SMA + P1 and P8 MECs (Fig. 3E). TrkB expression in MECs decreased postnatally, and TrkC was consistently expressed in MECs from E16 to P8. Expression of neurotrophin receptors in the E16 and early postnatal SMG was similar to our observations in non-irradiated human SMGs (Fig. 2F), in which peripheral nerves expressed NGFR and TRKA, and MECs expressed TRKA, low levels of TRKB and TRKC, and no notable expression of NGFR. Neurotrophins (NGF, NTF3 and NTF4/5) were mainly expressed in mouse and human by pericytes and MECs, which are both smooth muscle-like cell types, but they were more strongly expressed in MECs in the mouse SMG.

These observations support that murine SMG can be used to investigate neurotrophin signaling that occurs between multiple cell types in normal salivary gland function and development. The expression pattern of neurotrophin genes at E16 suggests that neurotrophin signaling likely mediates both autocrine and paracrine interactions among MECs, pericytes, nerves, stromal, and basal duct cells, with MECs being the main source of neurotrophin ligands in mouse, whereas pericytes, MECs and basal duct cells are the main source in humans (Fig. 3F, Supplementary Fig. 4).

### NGF correlates with late MEC differentiation whereas NTRK2 and NRTK3 mark early MEC development

**Fig. 4:**
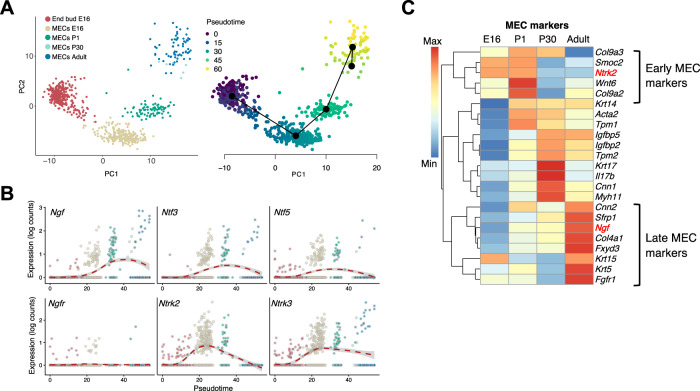
NGF correlates with late MEC differentiation whereas NTRK2 and NRTK3 mark early MEC development. **A** PCA plots of MECs and endbud cells from scRNAseq data. Left plot is colored by developmental stage and right plot by pseudotime score. **B** Expression of neurotrophin receptors across pseudotime in endbud cells and MECs. **C** Heatmap showing MEC genes that are differentially expressed between developmental stages.

The fact that neurotrophin genes appeared to be temporally regulated in MECs during development and that NTRs were strongly upregulated in MECs post-IR led us to hypothesize they could be involved in MEC differentiation. We performed pseudotime analysis using Slingshot^28^ to bioinformatically sort MECs according to their predicted developmental state. Because endbud cells from E16 give rise to MECs, we specifically extracted the scRNAseq data from E16 endbuds and MECs from subsequent developmental stages and re-clustered them (Fig. 4A). PCA of endbud and MEC clusters showed clear separation of cells by their developmental stage, and pseudotime analysis accurately sorted cells in order from E16 endbud to adult myoepithelial cells (Fig. 4A, B). When we evaluated expression of neurotrophins and their receptors in MECs across pseudotime, we observed a clear upwards trend showing the progressive increase in expression of *Ngf, Ntf3, Ntf5*, and *Ntrk3*, all of which peaked in adult MECs, whereas *Ntrk2* was highest in MECs at E16 and decreased in expression at later stages of development (Fig. 4B). Only a subset of E16 endbud cells and early MECs expressed *Ngfr*, whereas *Ntrk1* was not detected in MECs by scRNAseq.

Differential gene expression analysis in MECs across developmental stages confirmed that *Ngf* correlated with late MEC differentiation and *Ntrk2* with early MEC development (Fig. 4C, S4A). Among the genes that mark early MEC development (E16-P1), we also identified *Col9a2, Col9a3, Smoc2*, and *Wnt6*, whereas late MEC development (P30-adult) was characterized by elevated expression of *Il17b, Cnn1, Myh11, Fgfr1, Krt5, Krt15, Cnn2, Sfrp1, Col4a1*, and *Fxyd3* (Fig. 4C, Supplementary Fig. 5A). The expression of *Acta2* and *Krt14*, which are often used as bona fide MEC markers, were comparable across postnatal stages and only relatively low expressed at E16. In contrast, *Cnn1 and Myh11* were more strongly expressed in late development along with *Il17b* and *Krt17*.

Having identified *Cnn1* as a highly expressed and specific marker of postnatal MECs, we used multicolor in situ analysis (RNAscope) of *Cnn1* along with probes for *Ntrk1–3* and *Ngfr*, to confirm the expression of NTRs in MECs in postnatal SG (Supplementary Fig. 5B). *Ntrk3* was consistently expressed in MECs, *Ntrk2* was expressed in MECs at P1, and *Ngfr* was primarily observed in what appeared to be basal duct cells and ionocytes (Supplementary Fig. 5B), consistent with our previous observations in human. *Ntrk1* was barely detectable in mouse SMG. Note that some expression of NTRs is also expected along surrounding nerves (not shown). A subset of the genes identified here were used in downstream experiments as surrogates for MEC differentiation.

### NGF increases, whereas a pan-TRK inhibitor decreases markers of MEC and acinar differentiation in E16 SMG organ culture

**Fig. 5:**
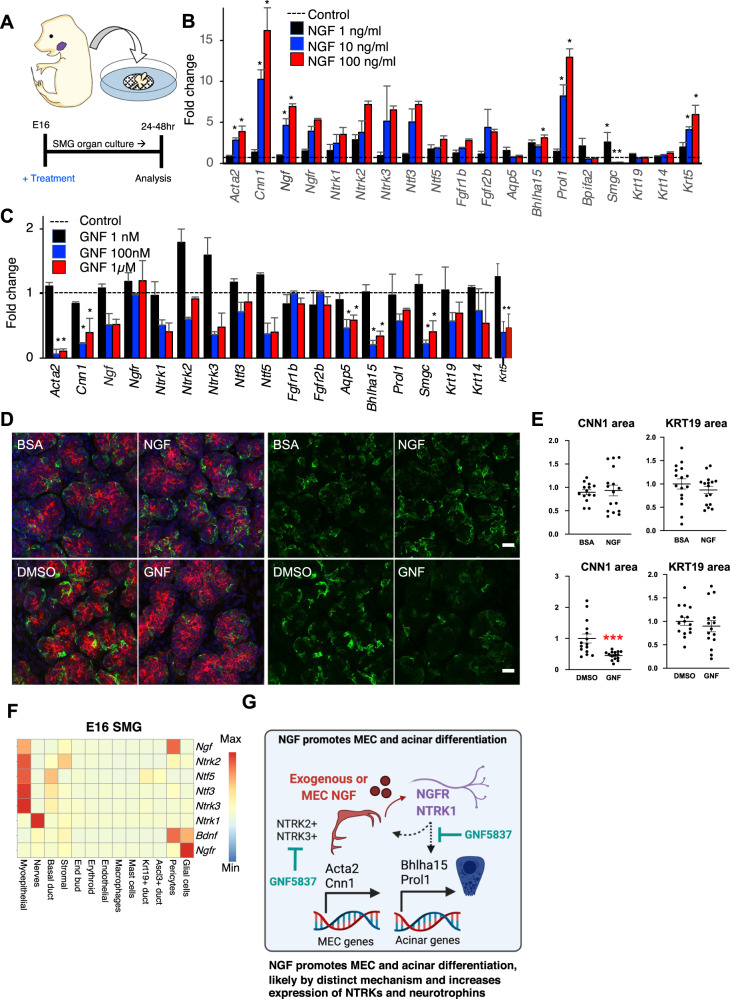
NGF promotes MEC differentiation in SMG organ cultures. **A** Experimental setup. Organ culture of E16 mouse SMG treated with NGF or GNF5837 for 24 h. **B**, **C** PCR results showing fold change gene expression of selected acinar, MEC, and duct genes 24 h post-treatment with NGF or GNF5837 at multiple doses. Statistical significance is represented by stars (*n* = 4 per group, *p* < 0.05; Two-way ANOVA with Dunnet’s correction for multiple comparisons). Error bars represent standard error of the mean. **D**) Immunofluorescence staining for MECS (CNN1, Green), developing endbuds/ducts (KRT19, Red) in E16 SMG collected 48 h after treatment with NGF (100 nM) or GNF (1 µM). Panels on the left show composite images while panels on the right show single-channel images for CNN1 staining. Scale bar = 20 µm. **E** Quantification of CNN1 + area and KRT19 + area from **D**. Dots represent individual areas analyzed from each gland where at least 5 random images per gland were analyzed. Stars denote statistical significance (students *t*-test, *p* < 0.001, *n* = 3 glands per group). The horizonal line and error bars represent the mean +/− SEM. **F** Heatmap of expression of neurotrophin signaling genes in scRNAseq from E16 SMG. **G**) Proposed mechanism for NGF induction of MEC and acinar differentiation via NTRK1 in nerves and glial cells.

In order to explore a causative role for NGF signaling in MEC differentiation, we used ex vivo cultures of developing SMGs and gain- and loss-of function experiments. We treated ex vivo organ cultures of E16 mouse SMG with three different doses of either recombinant human NGF (1, 10 and 100 ng/ml) or the chemical PanTRK inhibitor GNF5837 (GNF, at 10, 100 nM or 1 µM), which inhibits TrkA, TrkB and TrkC signaling, to stimulate or inhibit neurotrophin receptors for 24 h (Fig. 5A–C). We used E16 SMG because MEC differentiation is occurring at this timepoint and explants can be evaluated by qPCR using the developmental markers we identified in the scRNAseq data^17,27^.

NGF treatment resulted in a dose-dependent upregulation of *Acta2* and the late MEC differentiation markers *Cnn1* and *Krt5*, but no changes in *Krt14*, which is expressed throughout MEC development and by basal duct cells (Fig. 5B). Interestingly, NGF also upregulated expression of *Ngf*, *Ntf3*, *Ntf5*, *Ngfr*, and *Ntrk1–3*, suggesting a feedforward mechanism and positive regulation of other neurotrophin signaling genes. Surprisingly, treatment with NGF also increased expression of the early acinar marker gene *Bhlha15* and the serous secretory marker *Prol1*, but it reduced the expression of the embryonic mucous gene *Smgc* in a dose-dependent manner (Fig. 5B). Conversely, the panTRK inhibitor GNF downregulated expression of both MEC and acinar genes at 100 nM and above (Fig. 5C). Both NGF and GNF5837 downregulated the expression of the embryonic mucin gene *Smgc*, suggesting a balance of neurotrophin signaling may also influence serous and mucous acinar differentiation. In addition, expression of *Aqp5*, an acinar water channel, was not increased by NGF, but was reduced by panTRK inhibition, suggesting its expression may be regulated via TrkB or TrkC signaling. The specific roles of neurotrophins in acinar differentiation remains to be determined.

Evaluation at the protein level using immunofluorescence showed a decrease in the CNN1 + area after treatment with 1 µM GNF (Fig. 5D, E) but no differences were observed after NGF treatment. These observations combined suggest a direct role of NGF signaling in promoting expression of MEC and acinar differentiation genes. However, given that MECs are the primary source of endogenous NGF at this time point, and that the canonical NGF receptors (*Ngfr* and *Ntrk1*) are differentially expressed by nerves and glial cells (Fig. 5F), we propose that the regulation and induction of MEC and acinar genes by NGF is likely to occur indirectly via neuronal-dependent mechanisms (Figs. 3F, 5G).

### Dysregulation of neurotrophin receptors in MECs is associated with abnormal morphology and expression of KRT19

**Fig. 6:**
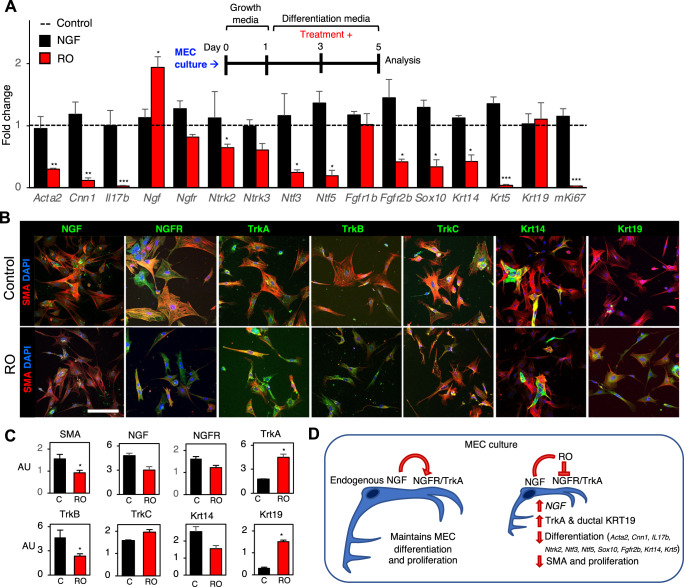
Dysregulation of neurotrophin receptors in MECs is associated with abnormal MEC differentiation and morphology. **A** Experimental setup of murine MEC culture treated with exogenous NGF or RO for 48 h. PCR results showing fold change gene expression of selected MEC and duct genes 48 h post-treatment with NGF or RO (*n* = 4 per group). Statistical significance (*p < 0.05; **p < 0.01; ***p < 0.001; Unpaired t-test compared to each control). **B** Immunostaining of MEC culture treated with RO for 48 h, with antibodies to NGF, neurotrophin receptors, KRT14 and KRT19 (all Green). All cultures were stained with SMA (red) and DAPI (blue). Scale bar = 150 µm. **C** Relative quantitation of MEC immunostaining from **B**, normalized to nuclei staining. Data is shown as mean +/− SEM. Two-tailed unpaired *t*-test was used. **D** Summary of NGF signaling in MEC culture.

To determine whether NGF signaling directly regulates a MEC differentiation program, we performed our gain and loss of function experiment in a MEC-enriched culture system using primary cells from postnatal mouse SMG (P2) (Fig. 6A, Supplementary Fig. 6). This system was developed using a modified version of a previously reported protocol for culture of MECs from lacrimal glands^29^. This culture enriches for MECs on collagen IV coated dishes and they are identified by their distinctive stellate morphology and co-expression of SMA (*Acta2*), and Cnn1 along with Krt14, FGFR1 and ColIV (Fig. 6B, Supplementary Fig. 6A).

The MEC cultures used in this study were screened for the expression of markers for various cell types such as myoepithelial (*Acta2*, *Cnn1* and *Vim*), acinar (*Aqp5* and *Prol1*), neuronal (*Tubb3*), endothelial (*Pecam1*), and mesenchymal (*Vim*) cells by qPCR analysis in comparison to intact postnatal gland. This MEC culture was enriched for cells expressing markers for MEC when compared to intact postnatal SMG (Supplementary Fig. 6B). There was some enrichment for tubulin III, a marker for neuronal cells and vimentin, which is expressed by both the mesenchymal and MECs. Importantly, MECs in primary culture express NGFR and TrkA, which is different from their low expression in vivo, and suggests they behave in culture as if they are responding to damage or stress, similar to human-IR MECs, and they show robust expression of NGF in brightly stained vesicles (Fig. 6B). MECs also expressed TrkC and had lower expression of TrkB.

Treatment of P2 MEC cultures with exogenous NGF (up to 100 ng/ml) for 48 h did not result in significant expression changes in any of the evaluated genes by qPCR (Fig. 6A). This is not surprising given that MECs have already differentiated at P1 and their abundant expression of NGF (Figs. 3C, 6B) may possibly obviate the effect of exogenous NGF. To specifically evaluate if endogenous NGF would impact MEC differentiation via NGFR, we used the NGF inhibitor, Ro-082750 (RO), which preferentially inhibits binding of NGF to NGFR. Inhibition of endogenous NGF signaling with RO downregulated both early and late MEC differentiation genes including *Acta2, Cnn1, Il17b, Ntrk2, Ntf3, Ntf5, Krt14*, and *Krt5* (Fig. 6A, B), and decreased expression of *Mki67*, a marker of cell proliferation, but upregulated *Ngf* itself.

In support of these changes in gene expression, we immunostained MEC cultures treated with and without RO (Fig. 6B, C). RO-treated MECs appeared smaller and less spread out than control cultures. We quantified the immunofluorescent staining intensity normalized to DAPI (Fig. 6C) and observed a reduction in SMA and TrkB staining, whereas TrkC, NGF, NGFR, and Krt14 were not significantly altered. In contrast, staining for TrkA and the duct marker Krt19 was significantly increased. qPCR of MECs treated with RO for 6 days in culture further confirmed the significant upregulation of *Krt19* (not shown).

The co-expression of TrkA and NGFR in MECs was only observed in in vitro culture and post-IR in human MECs, supporting that upregulation of NGF signaling could be a general response to stress that is maintained chronically. This is supported by the unexpected and abnormal increased expression of TrkA and Krt19 in RO-treated MECs, which could reflect both a general response to stress to upregulate NGF signaling and the plasticity that occurs in many cells as a result of stress^30^ (Fig. 6D). Thus, we next aimed to investigate whether there was evidence of stress-induced plasticity in MECs post-IR in humans.

### Upregulation of NTRs in human MECs post-IR is directly associated with abnormal duct-like characteristics

**Fig. 7:**
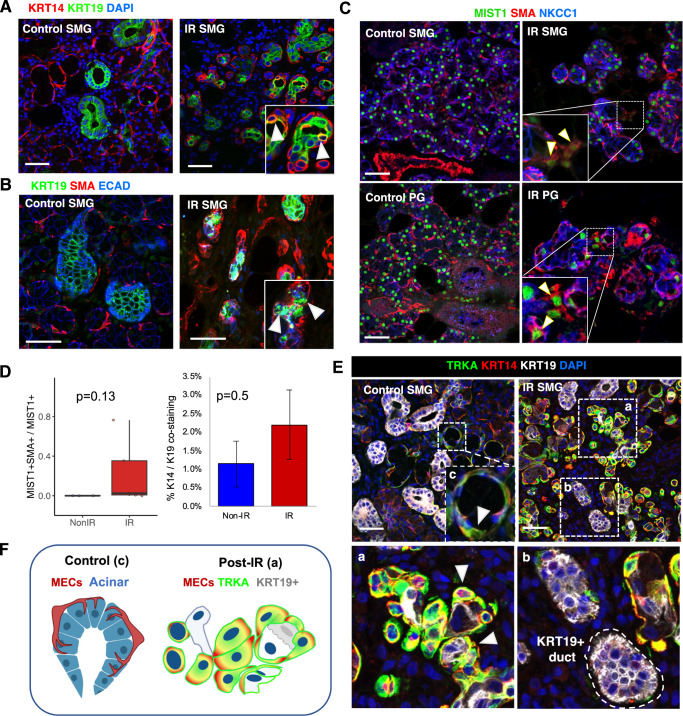
Upregulation of NTRs is directly associated with abnormal morphology and co-expression of MIST1 and KRT19 in MECs post-IR in humans. **A** Control and IR SMG stained for KRT14 (red), KRT19 (green), and DAPI (blue). White arrows point at cells with overlap between KRT14 and KRT19. Scale bars, 50 µm. **B** Control and IR SMG glands immunostained for KRT19 (green), SMA (red), and E-cadherin (blue). **C** Control and IR SMG and PG glands stained for MIST1 (green), SMA (red), and NKCC1 (blue). White arrows point at cells with overlap between SMA and MIST1. Scale bars, 50 µm. **D** Quantification of double-positive MIST1+/SMA+ cells normalized to number of MIST1+, and %SMA/K19 co-localization from IF staining (*n* = 3 PG IR, 2 PG non-IR, 4 SMG IR, 1 SMG non-IR). **E** Immunostaining of human SMG for KRT14 (Red), KRT19 (white), and TRKA (green). Delineated areas are expanded and denoted by labels ‘a’, ‘b’ and ‘c’. White arrows point at representative MECs. All scale bars, 50 µm. **F** Representation of morphological differences between control and IR MECs.

To search for supporting evidence that stress-induced plasticity may occur in human MECs post-IR, we performed immunostaining for established differentiation markers of MECs (SMA + KRT14+), luminal ducts (KRT19), and acinar cells (MIST1 + NKCC1+). Immunostaining showed the exclusive localization of these markers in control glands but identified rare populations of cells that were double positive for KRT14 + KRT19+ or SMA + KRT19+ (Fig. 7A, B), and cells co-expressing MIST1 + SMA+. Double-positive MIST1 + SMA+ cells were extremely rare even in IR glands (1.09% ± 0.75%, *n* = 3) but two IR biopsies showed 35–79% co-localization (Fig. 7C, D, Supplementary Fig. 7). The absolute number of double positive KRT14 + KRT19+ cells was difficult to quantify due to both markers showing cytoplasmic localization. Nonetheless, we saw a numerical increase in % area showing co-staining for both markers albeit it was non-significant (Fig. 7D). TRKA seemed to be exclusively upregulated in MECs that were double positive or adjacent to KRT19+ cells (Fig. 7Ea), but it was not expressed in KRT19+ ducts (Fig. 7Eb). A closer inspection also revealed that MECs of control glands had a stellate morphology with thin processes that wrapped around MIST1-positive acinar cells (Fig. 7Ec), which were KRT19-negative, while MECs post-IR showed a duct-like morphology and overlapped with or surrounded KRT19+ cells (Fig. 7Eb).

Our data combined supports an association between upregulation of neurotrophin signaling and stress-induced plasticity in MECs. Given that MEC plasticity can regenerate both acinar and ductal cells in preclinical models^7^, our findings put forward neurotrophin signaling as a new mechanism that could be harnessed to modulate MEC differentiation in a regenerative context. Further investigation is warranted to determine the essentiality of NTRs to modulate MEC plasticity and to address whether altering the balance and levels of specific neurotrophins could prevent or reverse the chronic IR damage that leads lack of acinar regeneration.

## Discussion

Cellular differentiation and plasticity are recognized as major interrelated mechanisms involved in normal development and regeneration of salivary glands^4,7^, and both can be triggered as a response to stress^8,30^. How these processes are disrupted in IR salivary glands where regeneration doesn’t occur remains unknown and is a topic of significant clinical interest. First and foremost, our study delineates the transcriptional changes in the dysfunctional PG and SMG and provides many targets for future research into mechanisms of salivary regeneration. We highlight multiple pathways that are altered in IR glands, including mechanisms involved in fibrosis, inflammation, and neurotrophic signaling. Among these pathways, neurotrophin signaling via NGF is a potential candidate for therapeutic targeting because it showed to be upregulated in abnormal MECs post-IR. This is of interest because of the ability of MECs to regenerate acinar cells^7^. For instance, MECs also have the highest expression of *Nrtn* according to scRNAseq analysis compared to all other cell types (data not shown) and NRTN was shown to preserve salivary function in irradiated SG of mice and minipigs when administered via gene therapy^12^. Furthermore, NRTN promoted regeneration in irradiated organ cultures via muscarinic receptors and EGFR signaling^10,31^. This information suggests that using a more directed approach that targets MECs may result in additional protective effect of increased MEC NRTN.

We discovered several abnormalities in human salivary MECs post-IR, including their aberrant localization, morphology, and expression of the acinar marker MIST1 and the ductal marker KRT19, along with upregulation of NGFR and NTRK1, 2, and 3. This is of considerable interest given that MECs have been shown to regenerate acinar cells upon severe stress in preclinical models, and it is feasible that dysregulation of neurotrophin signaling may impair their function. However, it is unclear whether these abnormal MECs co-expressing markers of different cell types reflect residual plasticity in an attempt to regenerate the gland, or cells that have failed to differentiate and became dysfunctional. Their isolation and further study are warranted. Reduced MEC differentiation measured by a decrease in expression of *Cnn1* and *Acta2* also precedes the progression of in situ ductal carcinoma to invasive disease in mammary gland^32–34^; thus, the clinical impact of targeting MEC differentiation may expand beyond SG regeneration.

NGF and NGFR have also been linked with lung, liver, and conjunctival fibrosis^35–38^ where expression of NGFR often correlates with severity of the disease^35^. Thus, further investigation in the role of NGF in IR-induced salivary fibrosis is needed. On the other hand, NGF also accelerates wound healing and closure in rodents^39,40^. These contrasting functions of NGF are influenced by the cell type-specific expression of NGFR and NTRK1 in distinct populations, and the coordinated action of these cell types. In models of hepatic fibrosis, NGFR was found to be expressed by stellate cells which underwent apoptosis upon stimulation with NGF^41^, whereas in skin NGFR and NTRK1 are found specifically in keratinocytes, nerves, and fibroblasts^42^. In our study, NGFR and NTRK1 were expressed in neuronal cells in both the developing mouse SMG and healthy human SG, suggesting that paracrine nerve stimulation could be responsible for the effects of NGF in development. Our MEC-enriched culture suggests that in the postnatal gland, changes in neurotrophin signaling may lead to alterations in MEC markers, but in this context, it is difficult to determine whether autocrine or paracrine mechanisms are responsible, given that many cell types expressed both NTRs and their ligands, including pericytes, MECs, ionocytes, and basal duct cells. Understanding these nuances in the spatial-temporal regulation of neurotrophin signaling genes will be an instrumental next step in order to attain the desired effect upon targeting NGF/NTRs. Given the strong association between fibrosis and chronic salivary dysfunction post-IR and the role of NGF in modulating expression of MEC genes, the benefit of targeting neurotrophin signaling may be two-fold. Furthermore, selective agonists and inhibitors for NTRs are available thus facilitating further investigation into their clinical applications in SG regeneration.

Our bioinformatic analysis also revealed FGF, IGF, and Notch signaling pathways among the predicted upstream regulators compromised by IR. Due to the known roles of these pathways in salivary gland function, development, and repair, they too warrant further investigation. For instance, IGF has demonstrated to both preserve and restore secretory function in irradiated glands of mice by promoting cell cycle arrest and DNA repair^43^, or by activation of aPKCζ^44^. FGF is required for salivary gland development and progenitor differentiation^31,45–49^. How these pathways influence MEC plasticity in the gland, or whether they contribute to the development of fibrosis post-IR remains to be determined.

In summary, we present that neurotrophin signaling is one of the major pathways dysregulated by IR in salivary glands involving numerous cell types, and that changes in neurotrophin signaling may affect MEC differentiation, plasticity, or both. We propose that MECs undergo a process of stress-induced plasticity linked to the upregulation of neurotrophin signaling in response to IR. This may help explain the lack of regeneration post-IR in at least two ways: first, by preventing the ability of MECs to act as precursors for regeneration of acinar and duct cells as previously described^7^, and second, by disrupting the balance of neurotrophin signals to other cell types (i.e. glia and nerves) that may also be required for regeneration.

Our study focused on NGF and did not address the functions of other MEC-produced neurotrophins such as Ntf3 and Ntf5. It’s possible that the balance of neurotrophin signaling from multiple neurotrophins may be critical to the regeneration of MECs into acinar and duct cells. In addition, we did not elucidate specific roles for other NTRs which are also upregulated post-IR. The aberrant expression of NGFR and TRKA in MECs after IR were also associated with increased expression of TRKB and TRKC in MECs. There are likely autocrine roles for MEC derived neurtrophins to influence MEC differentiation. Furthermore, our data suggests that neuronal cells, both glia and nerves, may be important for development of MECs in vivo. Further investigation of the role of neuronal-MEC interactions during SG development and function remain to be investigated.

## Methods

### Reagents and materials

A list of all reagents, materials, and equipment used in this study is provided in Supplementary Tables 4–6.

### Collection of human specimens

Biopsies from control and irradiated PG and SMG were collected from volunteers undergoing surgery for head and neck cancer at the Princess Margaret Hospital (Toronto, CA). The Institutional Review Boards approved all research procedures and the study participants gave written informed consent (Protocol #UHN 2016–0486 & REB #11–0988-CE). Upon resection, samples were placed in a sterile 0.5 ml tube and shipped on ice overnight to NIDCR for downstream analysis by RNAseq and salisphere culture. Samples were weighted and measured upon arrival. When possible, each specimen was subcut into multiple pieces to process for RNA extraction and FFPE sectioning.

### Mice

All mice were maintained and treated according to guidelines approved by the National Institute of Dental and Craniofacial Research and National Institutes of Health Animal Care and Use Committee (ASP#10–2022).

Timed-pregnant ICR females were purchased from Envigo at gestational day E9. From this point onwards mice were cared for and maintained at the NIDCR Veterinary Resource Core in accordance with institutional and IACUC guidelines. All mice were fed ad libitum and kept under 12-h light/12-h dark cycle. Pregnant females used for collection of mouse embryos were housed in pairs while those required for collection of newborn pups were individually housed to prevent overcrowding. Pregnant females were euthanized in a CO_2_ chamber for 6 min followed by cervical dislocation according to IACUC recommendations. Mouse embryos were collected from ICR pregnant females at embryonic day E16 for SMG organ cultures and immunostaining, and newborn pups were collected at P1, P2, and P8 for immunostaining and isolation and culture of MECs. Embryos and newborn pups were euthanized by decapitation.

### Immunohistochemistry

SGs were fixed in 4% paraformaldehyde overnight at 4 °C and dehydrated with 70% Ethanol prior to paraffin embedding. 5 µm sections were deparaffinized with xylene substitute for 10 min and rehydrated with reverse ethanol gradient for 5 min each. Heat-induced antigen retrieval was performed using a microwave maintaining sub-boiling temperature for 10 min in a pH 6.0 Citrate Buffer (#21545, EDM Millipore, Darmstadt, Germany). Sections were washed for 5 min with 0.1% Tween20 (Quality Biological, Inc) in PBS 1× (PBST). Mouse tissues were stained using the M.O.M.® (Mouse on Mouse) Immunodetection Kit (Vector Laboratories, Burlingame, CA) to block non-specific sites for 1 h at room temperature followed by overnight incubation with primary antibodies at 4 ^o^C. Tissue sections were washed three times for 5 min each with PBST and incubated in secondary antibodies and nuclear stain (Hoechst (Thermo Fisher Scientific, Marietta, OH)) at room temperature for 1 h. Coverslips were mounted with Fluoro-Gel (Electron Microscopy Sciences, Hatfield, PA), and imaging was performed with a Nikon A1R confocal system. Quantitation of number of MIST1+/SMA+ cells was performed manually and normalized to total number of MIST1+ nuclei. For analysis of KRT19/KRT14 co-localization, we applied the co-localization test from ImageJ with default settings and report the %volume of co-localized pixels between the two channels. In both cases, at least random areas were imaged from each biopsy (40×, 10 µm stack, 1 µm interval) using the Nikon A1R confocal microscope. Quantitation of staining area was done on Maximum Projections with automatic threshold and stack histogram using ImageJ. Students *t*-test was used to calculate statistical significance. A detailed list of the antibodies’ provenance and dilutions used in our study are provided in Supplementary Table 4.

### In situ hybridization

Freshly dissected salivary glands from P1 and P30 mice were collected in 200 µl eppedorf tube and washed in 1× PBS RNAse free solution. All tissue was maintained in RNAse free conditions during this protocol and all tools were cleaned with 70% ethanol and wiped before use. Freshly prepared tissue was placed in 4% PFA for no longer 36 h and sent to Advanced Cell Diagnostics (ACD) with for RNA in situ hybridization with respective probes. Sample were also accompanied by dehydration pockets to remove mosture from prepared slides and slide were immediately placed in 4oC prior to imaging. Specific probe sequences are proprietary and generated with RNAscope® technology by ACD.

### Salisphere culture (human specimens)

Salivary gland specimens were enzymatically dissociated in 5 ml of digestion cocktail containing Collagenase II (100 mg/ml, Gibco) and Hyaluronidase (50 mg/ml, Sigma) with 6.25 nM CaCl2 in 1% HBSS-BSA. Glands were manually minced and then further dissociated for 30–40 min at 37 ^o^C in a 15 ml gentleMACS C tube in a Miltenyi gentleMACS Octo Dissociator (Miltenyi Biotech, Auburn CA). Following dissociation, 5 ml of RPMI media were added to the dissociated cells and centrifuged at 1100 rpm for 10 min. Cells were resuspended in RPMI 1640 w/L-Glutamine with 5% PenStrep (Gibco, USA) and washed twice with RPMI. Cells were passed through 70 µm filters between centrifugation steps. Cell concentration was determined with a Cellometer (Nexcelom Biosciences) and 200,000 cells were plated in in low-adhesion 24-well plates (Costar) in 400 µL sphere media. Sphere media consisted of DMEM-F12/1% penicillin-streptomycin, 10 μg/mL ITS, 1% N2, (Gibco); 1 μM dexamethasone, 20 ng/mL FGF2, 20 ng/mL EGF, 100 ng/mL FGF10, 100 ng/mL recombinant mouse stem cell factor, SCF (R&D Systems); 10 nM carbachol (Cch C4382, Sigma); and 5 μM Y27632,a ROCK-inhibitor (Sigma). Salispheres were cultured for up to 10 days and an additional 50 µL of fresh media was added to replenish the culture medium every two days. At 3, 5, 7, and 10 days in culture, salispheres were analyzed using an EVOS microscope at 4× and 10× objectives to count number and size of generated spheres, respectively.

### RNA isolation and qPCR

Total RNA was extracted to evaluate quality prior to RNAseq using the RNAqueous-4PCR kit and DNase removal reagent (Ambion, Inc. Austin, TX). Only samples with RIN > 7 were submitted for RNAseq analysis. For qPCR analysis, cDNA (20 ng) was generated using SuperScript™ III First-Strand Synthesis System (ThermoFisher Scientific). qPCR was performed with iQ SYBR Green Supermix (Bio-Rad, 1708882) in a CFX96 Touch™ Real-Time PCR Detection System (Bio-Rad, 1855195). Melt curve analysis was used to verify the generation of a single amplicon. Expression levels were normalized by the delta-delta Ct method to the housekeeping gene Rsp29. Human samples were normalized to GAPDH. At least four biological replicates for each group were processed except when otherwise specified. A list of the primers used in this study are provided in detail in Supplementary Table 5.

### RNAseq

For RNAseq, cDNA libraries were prepared at the NIDCR Genomics and Computational Biology Core using the TrueSeq RNA Sample Preparation Kit (Illumina) from RNA samples with RIN > 7. The cDNA libraries were then sequenced on a NextSeq500 sequencer (Illumina) following the manufacturer’s protocol. The Grh38 reference genome (Genome Reference Consortium Human Build 38) was used for read alignment. The resulting counts matrix was imported into R for read count normalization and differential gene expression analysis. We used the EdgeR package in R Studio to import, filter, and normalize the data, followed by DeSeq2-Limma pipeline to identify IR-induced differentially expressed genes (DEGs) using a threshold of *p* < 0.05 and fold change larger than 2 as cutoff for significance.

### Import SG data from Tabula Sapiens

Data objects for the SG dataset were downloaded from the Tabula Sapiens and imported as SEURAT objects for analysis. The specimens were split by donor and re-clustered to reassign cell cluster labels consistent with our previous work and other salivary gland datasets. We used the standard integration pipeline provided by SEURAT. Differentially expressed genes (DEGs) in each cell type were determined using the ‘FindAllMarkers’ function, which uses a Wilcoxon Rank Sum statistical test for analysis. Only genes with adjusted *p*-values < 0.05 were considered as DEGs.

### Import scRNAseq SMG atlas

SEURAT-ready files containing scRNAseq from embryonic and postnatal murine SMG were downloaded from Hauser et al.^17^. These SEURAT objects were already annotated and clustered and thus were imported directly for downstream analysis. We extracted the data corresponding to E16, P1, and P30 glands, which were re-clustered and integrated using SCTransform and Integration pipeline from SEURAT. The new resulting clusters corresponded to the original annotations provided in the dataset. Differentially expressed genes (DEGs) in each cell type were determined using the ‘FindAllMarkers’ function, which uses a Wilcoxon Rank Sum statistical test for analysis. Only genes with adjusted *p*-values < 0.05 were considered as DEGs. We then compared our list of IR-induced DEGs in irradiated human SMG against the cell-specific DEGs in our integrated SEURAT object to predict possible enrichment in specific cell types.

### Ligand-receptor analysis

A database of curated ligand-receptor pairs was downloaded from ref. ^50^. We used scripted code in R to automate the search for expression of ligand and receptor genes within our dataset and leverage that information against the curated database. Only DEGs for each cell type were considered for this analysis. Plots were generated using the ‘circlize’ package in R.

### Trajectory analysis

To evaluate genes involved in MEC differentiation, we extracted MECs and E16 endbud cells from our integrated SEURAT object using the subset function. The new object was once again re-clustered as described above and Pseudotime was estimated using the slingshot algorithm^28^ within the Dynverse package^51^. Cells were sorted by their pseudotime score to evaluate the temporal expression of selected neurotrophin signaling genes in MECs.

### SMG organ culture

SMG were freshly dissected from E16 embryos using a stereo microscope (Leica Microsystems, Wetzlar, Germany) to remove the connective tissue capsules and septa surrounding the parenchyma. Each explant was culture on polycarbonate filters on top of 200 µl of DMEM (Dulbecco’s Modified Eagle Medium) containing 1% Penicillin-Streptomicin, vitamin c, and transferrin. NGF, GNF5837, or their corresponding vehicle controls were added directly into the media at the desired concentrations (1, 10, and 100 ng/ml of NGF and 1 nm, 100 nm, or 1 µM of GNF5837) within the first 2 h of culture. Glands were collected after 24 h for qPCR analysis. For whole-mount immunofluorescence, glands were cultured for 48 h and then fixed with ice-cold acetone:methanol (1:1) for 15 min at −20 ^o^C. Glands were incubated in 10% heat-inactivated Normal Donkey Serum, 1% BSA and 1% Mouse on Mouse blocking reagent (Vector Laboratories, USA) for 1 h at room temperature followed by overnight incubation with primary antibodies (anti-CNN1 (ab46794, Abcam, USA), and anti-Keratin 19 (Troma III, DSHB, USA) at 4 ^o^C. The glands were washed in PBS containing 0.1% Tween-20 (PBST) and incubated in secondary antibodies and DAPI nuclear stain at room temperature for 2 h. Glands were mounted on microscope slides (Fisherfinest, Fisher Scientific, PA, USA) with two 0.12 mm thick secureseal imaging spacers (Grace Bio-Labs, USA), coverslips and Fluoro-Gel mounting media (Electron Microscopy Sciences, Hatfield, PA). Five random areas were imaged from each of the three SMGs (40×, 10 µm stack, 1 µm interval) using the Zeiss 880 confocal microscope. Quantitation of staining area was done on Maximum Projections with automatic threshold and stack histogram using ImageJ. Students *t*-test was used to calculate statistical significance.

### MEC culture

SMG from at least six postnatal day 2 mice were collected, mined and dissociated in a 15 ml gentleMACS C tube with 5 ml of digestion enzyme using 0.575 mg/mL collagenase type II (Gibco, USA), 1 mg/mL hyaluronidase (Sigma, USA) and 6.25 mM CaCl_2_ (Quality Biological, USA) diluted in Hanks Buffer Salt Solution (HBSS) (Thermo Scientific, USA). Cell dissociation was performed in a Miltenyi gentleMACS Octo Dissociator using the manufacturer’s preset 37C_h_TDK_2 program. Following dissociation, HBSS buffer was added to the dissociated cells and centrifuged at 400 × *g* for 8 min. Cells were resuspended in 1 mL TrypLE Express enzyme (Thermo Fisher Scientific, USA) and incubated for 10 min at 37 ^o^C to dissociate further into single cells. HBSS buffer containing 1% BSA was added to the cells and centrifuged as described earlier. Cells were passed through 100 µm filter (Falcon, USA), centrifuged and washed again with HBSS buffer. Pellet was resuspended in smooth muscle cell growth media (Cell applications, Inc., USA) and strained through 70 µm filter (MACS Miltenyi Biotec, USA).

Cell count was determined with a Cellometer (Nexcelom Biosciences). Then, 50,000 cells were plated on 24-well BioCoat^TM^ Collagen IV multiwell plates (Corning, USA, #08–774–29) or 15,000 cells were plated on 8 well collagen IV coated 15-μ slide (Ibidi, Fitchberg, Wisconsin, USA, #50–305–885) in Smooth muscle growth media (Cell Applications Inc., San Diego, CA, USA, #311–500). After 24 h, media was removed and replaced with Smooth muscle differentiation media (Cell Applications Inc., San Diego, CA, USA, #300D-250) for the duration of the experiment. Treatment with 100 ng/mL NGF (R&D Systems) or 10 μM NGF inhibitor Ro-08–2750 (Tocris, USA #2272) was added after 3 days in culture and MECs were collected for qPCR or immunostaining after 48 h.

For qPCR, cells were lysed for RNA extraction using the RNAqueous Micro Kit (invitrogen, Thermo Fisher Scientific, Vilnius, Lithuania #AM1914) following the manufacturer’s instructions. For staining, MECs were fixed with 4% PFA for 20 min at room temperature or ice-cold acetone:methanol (1:1) for 10 min, washed and stored in 1× PBS until processing. Images were collected using 20× objective and 1 zoom magnification (5–11 slices of 0.3–1 µM thick). Maximum intensity projections were quantified by normalizing the fluorescence intensity of the protein of interest to that of the nuclei. Three experiments with at least 3 positions were quantified per treatment. Data is shown as mean +/− SEM. Two-tailed unpaired *t*-test was used.

### Reporting summary

Further information on research design is available in the Nature Research Reporting Summary linked to this article.

## Supplementary information

Supplementary Figures 1–7

Supplementary Data 1 - Differentially expressed genes.

Supplementary Data 2 - Pathway analysis

Supplementary Data 3- Upstream Regulator Analysis

Reporting Summary

## Data Availability

The RNAseq data used for the study are deposited to GeneExpression Omnibus (GSE206878). No cell lines, plasmids, or genetic tools were generated in this study.

## Code availability

Scripted analysis was performed using R (version 4.1.1). All code to download and process the bulk RNAseq data, as well as the single-cell RNAseq datasets from the SMG atlas and Tabula Sapiens has been deposited to a publicly available repository located in https://github.com/chiblya/IR_SalivaryGlands. Software used for analysis is reported in Supplementary Table 7, and all R packages and versions used for scripted analyses are listed in the GitHub repository. Further information and requests should be directed to and will be fulfilled by the Lead Contact Dr. Alejandro M. Chibly at martinez-chibly.agustin@gene.com.
