# Supplementary material for: Neurotrophin signaling is a central mechanism of salivary dysfunction after irradiation that disrupts myoepithelial cells
Source: NPJ Regen Med. 2023 Mar 25;8:17. doi: 10.1038/s41536-023-00290-7 (PMC10039923; doi:10.1038/s41536-023-00290-7)
Supplement: Supplementary file 1 — Supplementary Figures 1–7 [file 41536_2023_290_MOESM1_ESM.pdf]

Supplementary Figure 1

A

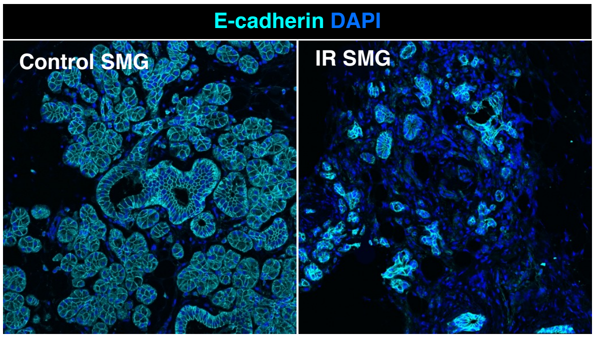

D

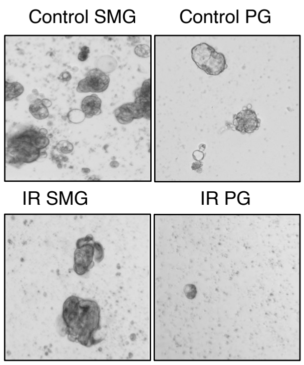

E

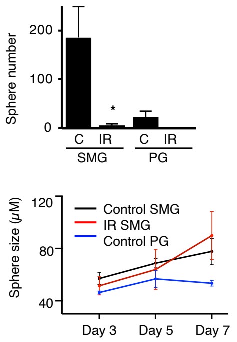

B

Salivary gland biopsies used for RNAseq

Sample ID	Sex	age	Cancer	Time from IR to surgery (months)	IR fractions	Estimated IR dose to salivary gland (Gy)
s04_PAR	M	69	buccal			
s06_PAR	M	78	skin			
s08_PAR	M	43	parotid			
s20_PAR	M	62	hard palate			
s25_PAR	M	56	buccal			
s26_PAR	M	70	skin			
s22_PAR_IR	F	66	tongue	212		Unknown
s35_PAR_IR	M	65	scalp	4	25	60
s37_PAR_IR	M	56	tongue	4	35	70
s38_PAR_IR	M	69	hypopharynx	4	35	70
s39_PAR_IR	F	62	pharynx	140	40	64
s04_SMG	M	69	buccal			
s07_SMG	M	70	scalp			
s09_SMG	M	60	larynx			
s12_SMG	M	62	hard palate			
s14_SMG	M	57	oral cavity			
s17_SMG	M	42	lip			
s23_SMG	M	53	tongue			
s01_SMG_IR	M	59	SCC	7	40	64
s02_SMG_IR	M	78	SCC	9		Unknown
s21_SMG_IR	F	66	tongue	212	33	66
s27_SMG_IR	M	62	SCC	5	35	70
s30_SMG_IR	F	77	Oropharynx	81	25	60
s31_SMG_IR	M	59	larynx	156	20	51

C

Clinical features of samples used for salisphere culture

	Control SMG			IR SMG			Control PG			IR PG		
	n	*Mean*	*SEM*	*n*	*Mean*	*SEM*	*n*	*Mean*	*SEM*	*n*	*Mean*	*SEM*
Age		63.31	3.09		62.14	2.878		68.93	4.20		78	6
IR Dose (Gy)		NA	NA		6380	317.6					5425	1175
Weight (mg)		407.20	82.51		257.1	45.75		315.00	61.00		426.7	54.57
Blood *		1.75	0.21		2	0.2344		2.64	0.13		3	0
Fibrotic tissue *	16	1.88	0.22	14	2.5	0.1738	14	2.21	0.19	3	2	1
Fat *		1.88	0.18		2.357	0.225		2.43	0.20		3	0
cell count		1.00E+07	1.83E+06		3.78E+06	1.12E+06		4.68E+06	1.28E+06		4.67E+06	3.30E+05
viability		73.67	3.82		69.92	4.799		69.80	4.42		75.1	17.7
Sphere count (d3)	11	167.20	54.99	8	12	7.351	10	25.40	12.75	3	0	0
Sphere count (d5)	16	179.50	51.08	12	14.18	9.814	14	22.88	11.98	3	0	0
Sphere count (d7)	10	141.40	46.51	6	6.833	6.833	9	12.56	8.72	0	NA	NA
Sphere count (d10)	9	151.00	41.84	8	3.5	3.5	9	16.00	7.05	0	NA	NA

* The presence of blood, fibrotic, and adipose tissue in biopsies was visually scored upon receipt using integer numbers between 1-3 for low, medium, and high levels, respectively.

Supplementary Figure 1. Clinical features of control and irradiated human PG and SMG biopsies collected for RNAseq analysis.
A) Immunostaining of human salivary glands for E-cadherin.
B) Table with description of SMG and PG biopsies collected for sphere culture.
C) Table with clinical description of samples collected for RNAseq analysis.
D) Representative images of sphere cultures from control and IR biopsies after 7 days in culture.
E) Bar graph shows the number of spheres after 7 days in culture. The number of replicates per group are as follows: SMG Control: 15, SMG IR: 11, PG control: 14, PG IR: 3. Star denotes significance (two-tailed t-test, $p<0.05$). Line graph shows the average sphere size at 3, 5, and 7 days in culture (number of replicates (n) = SMG Control (d3=7, d5=7, d7=6), SMG IR (d3=4, d5=3, d7=2), PG Control (d3=4, d5=4, d7=2)).

Supplementary Figure 2

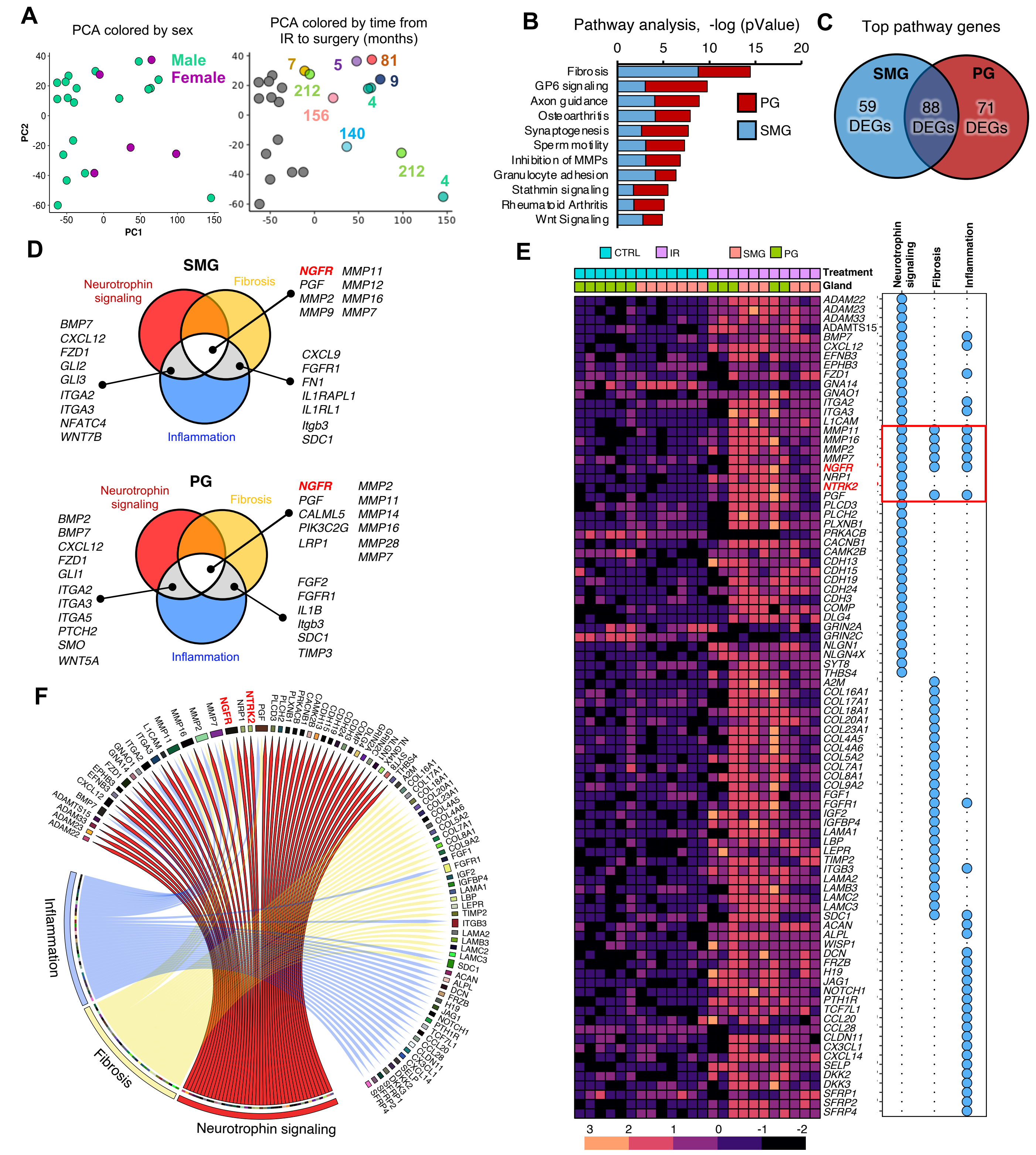

Supplementary Figure 2. Pathway and upstream regulator analysis of RNAseq data from control and irradiated human PG and SMG biopsies.

A) PCA plot of log-transformed CPM counts from bulk RNAseq analysis of human biopsies colored by sex or time to sample collection.

B) Results from Ingenuity Pathway Analysis (IPA) Software showing common pathways dysregulated in PG and SMG A combined score. was calculated by adding the –Log(p-val) for a given pathway in both glands.

C) Venn diagrams highlighting overlap between top-pathway-associated genes in IR-PG and IR-SMG.

D) Venn diagrams highlighting overlap between top-pathway-associated genes for each gland independently.

E) Heatmap showing 88 unique genes associated with top three dysregulated pathways in both glands.

F) Chord plot showing DEGs associated with the top dysregulated pathways in IR-PG and IR-SMG.

Supplementary Figure 3

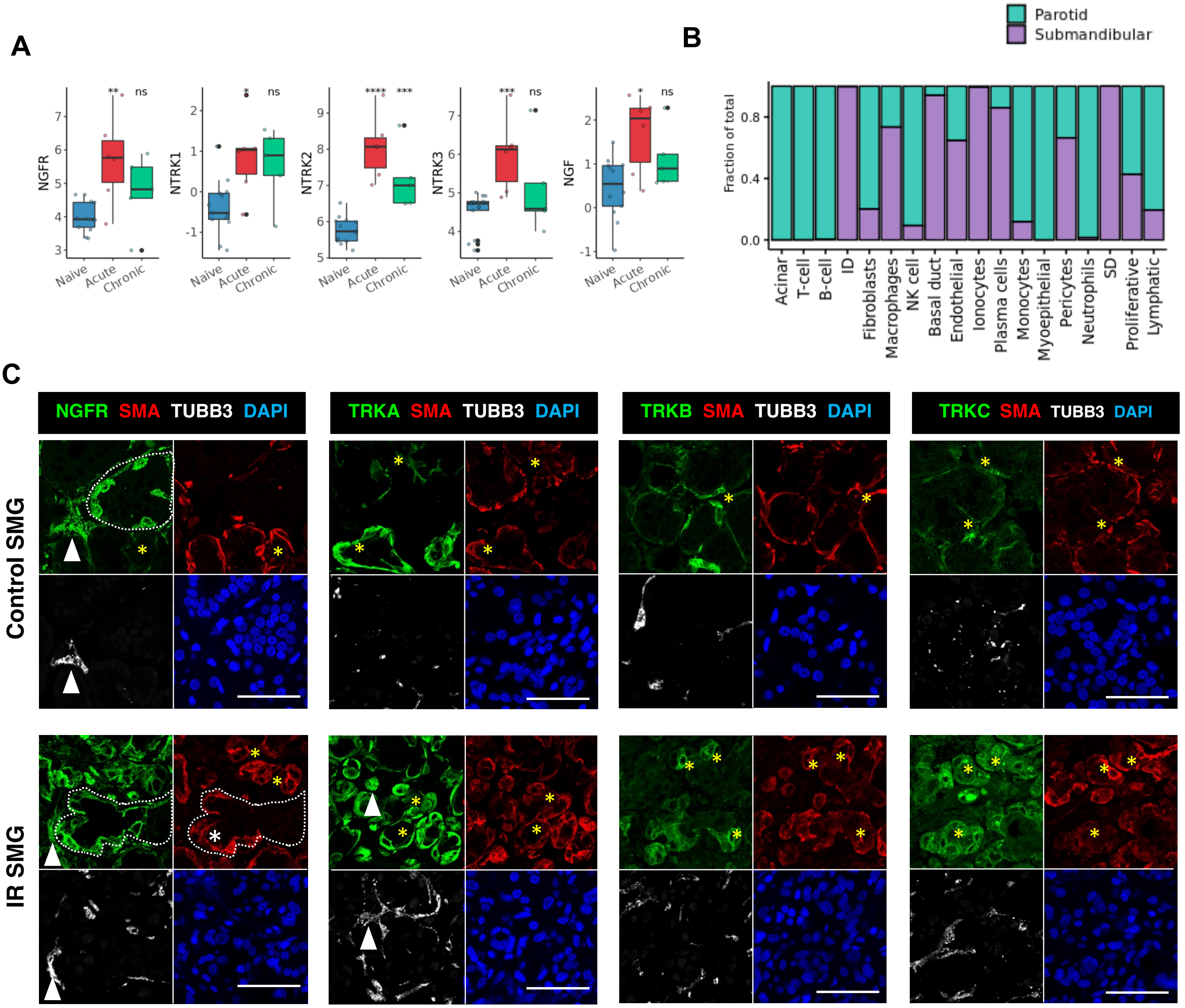

Supplementary Figure 3.

A) Box plots with expression of neurotrophin signaling genes in bulk-RNAseq data from human salivary glands. Acute = samples collected within a year of IR. Chronic = samples collected >1year post-IR. The box plot represents the median and interquartile range while the bar represents the minimum and maximum values.

B) Proportion of cells derived from parotid and SMG in Tabula sapiens scRNAseq data.

C) Immunofluorescent staining of human SMG for SMA, which labels MECs (red), Tubb3, which labels peripheral nerves (white), and neurotrophin receptors (green). White arrows point at nerves positive for NGFR. Yellow stars highlight representative MECs positive for neurotrophin receptors.

Supplementary Figure 4

Ligand-Receptor interactions involving neurotrophin genes in E16 SMG

FROM	TO	#	Ligand Receptor Pairs
MECs	Stromal	5	Ntf5→Ngfrap1, Ntf5→Ntrk2, Ntf3→Ntrk2, **Ngf→Maged1, Ngf→Ngfrap1**
MECs	MECs	4	Ntf5→Ntrk3, Ntf5→Ntrk2, Ntf3→Ntrk3, Ntf3→Ntrk2
MECs	Glial cells	4	Ntf5→Ngfr, Ntf3→Ngfr, **Ngf→Ngfr,** App→Ngfr
MECs	Nerves	3	Ntf5→Ngfrap1, **Ngf→Kidins220**, **Ngf→Ngfrap1, Ngf → Ntrk1***
Basal duct	MECs	2	Ntf5→Ntrk3, Ntf5→Ntrk2
Pericytes	Nerves	2	Ngf→Kidins220, **Ngf→Ngfrap1**
Basal duct	Glial cells	2	Ntf5→Ngfr, App→Ngfr
Nerves	Glial cells	2	Rtn4→Ngfr, App→Ngfr
Basal duct	Stromal	2	Ntf5→Ngfrap1, Ntf5→Ntrk2
Pericytes	Stromal	2	**Ngf→Maged1, Ngf→Ngfrap1**
MECs	Ascl3+	1	**Ngf→Sort1**
Pericytes	Ascl3+	1	**Ngf→Sort1**
Basal duct	Nerves	1	Ntf5→Ngfrap1
Krt19+ duct	Glial cells	1	App→Ngfr
Ascl3+	Glial cells	1	App→Ngfr
Pericytes	Glial cells	1	Ngf→Ngfr
Stromal	Glial cells	1	Rtn4→Ngfr

Supplementary Figure 4.
Table showing all predicted ligand-receptor interactions involving upstream regulator genes based on their expression in mouse MECs.

Supplementary Figure 5

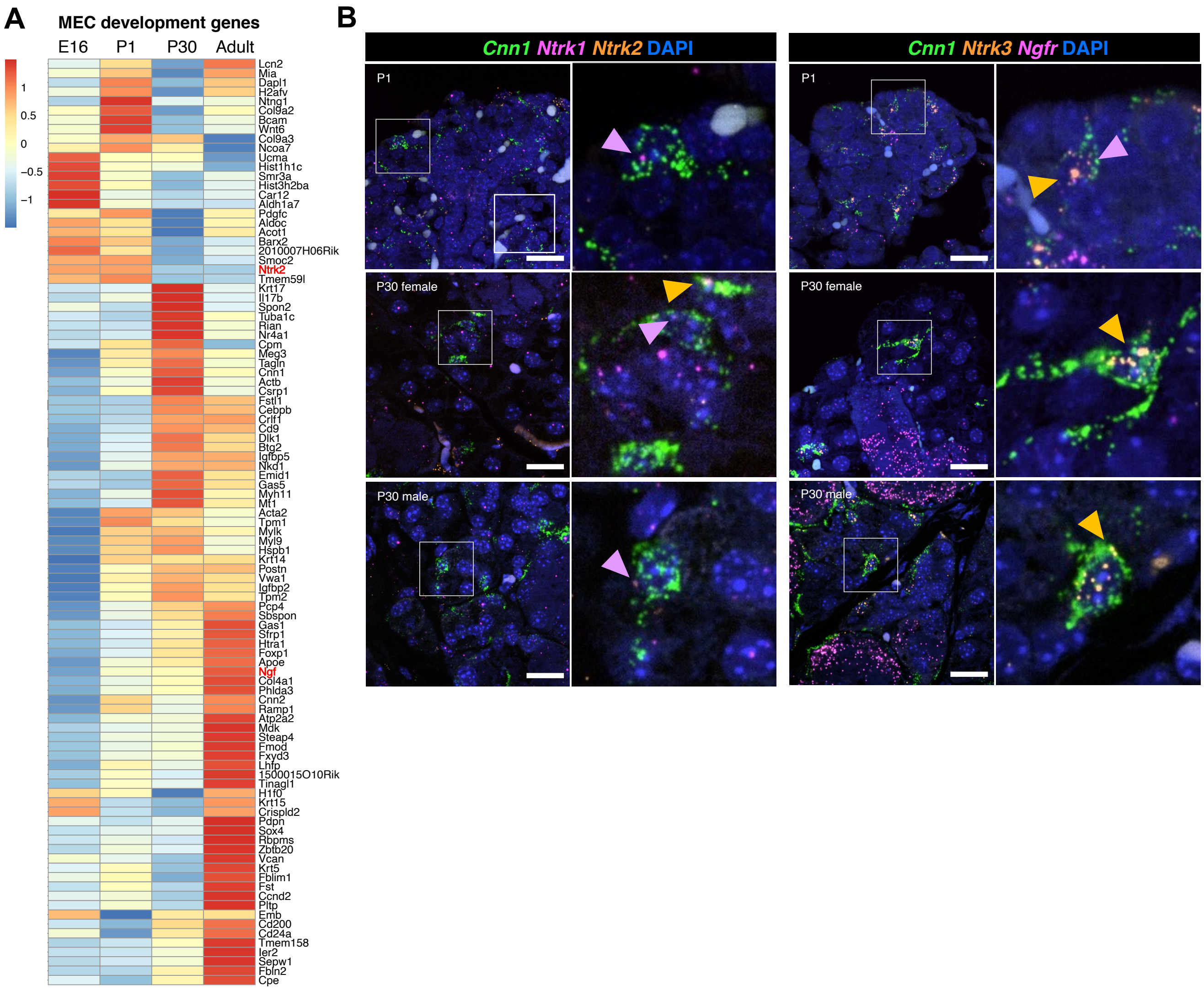

Supplementary Figure 5. Analysis of gene expression during murine MEC development

A) Heat map showing genes enriched in MEC during SMG development.

B) In situ analysis of NTRs. Arrowheads point at co-localization of the respective color-coded NTR with MECs.

Supplementary Figure 6

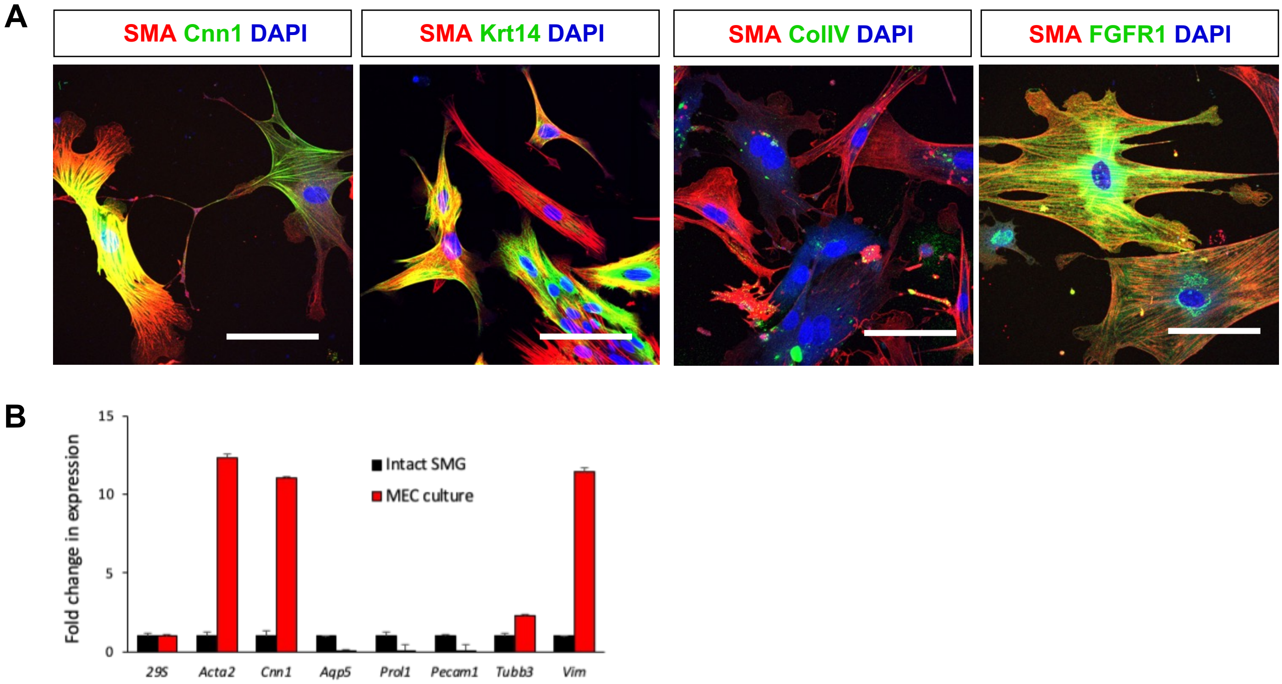

Supplementary Figure 6.

A) Immunofluorescence staining for smooth muscle actin (SMA; red) and Cnn1, Krt14, ColIV, and Fgfr1 as shown in the labels. Scale bar = 50um.

B) qPCR of cultured MECs vs intact SMG (P2) showing expression of selected genes. Normalized to *Rs29* and intact SMG.

Supplementary Figure 7

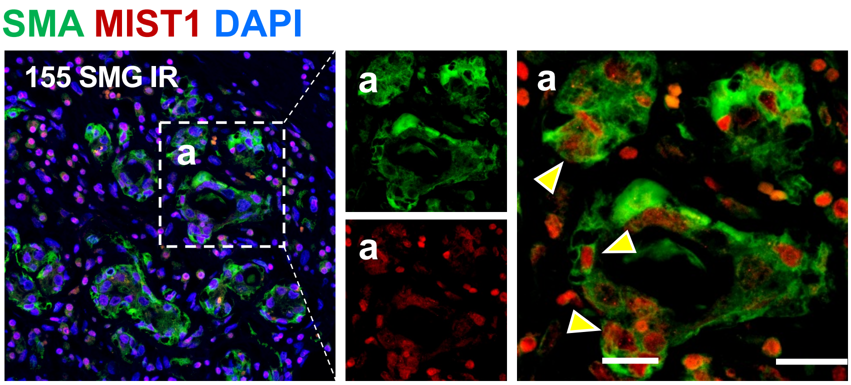

Supplementary Figure 7.
Immunofluorescence staining for smooth muscle actin (SMA; green), and MIST1 (red). Scale bar = 50um. The expanded region in a) shows unusual colocalization between SMA and MIST1.

Supplementary Table 1. Top 15 dysregulated pathways in IR-PG

Ingenuity Canonical Pathways	-log(p-value)	Genes
GP6 Signaling Pathway	9.43	CALML5, COL12A1, COL15A1, COL16A1, COL17A1, COL18A1, COL20A1, COL23A1, COL4A1, COL4A2, COL4A5, COL4A6, COL5A2, COL7A1, COL8A1, COL9A2, ITGB3, LAMA1, LAMA2, LAMA3, LAMB1, LAMB3, LAMC1, LAMC2, LAMC3, PIK3C2G
Hepatic Fibrosis / Hepatic Stellate Cell Activation	8.05	A2M, BAX, COL12A1, COL15A1, COL16A1, COL17A1, COL18A1, COL20A1, COL23A1, COL4A1, COL4A2, COL4A5, COL4A6, COL5A2, COL7A1, COL8A1, COL9A2, FAS, FGF1, FGF2, FGFR1, IGF2, IGFBP4, IL1B, LAMA1, LBP, LEPR, MMP2, **NGFR**, PGF, TIMP2
Synaptogenesis Signaling Pathway	7.3	ADCY3, ADCY5, APOE, CACNA2D1, CACNB1, CALML5, CAMK2B, CDH13, CDH15, CDH17, CDH19, CDH24, CDH3, COMP, DAB1, DLG4, EFNB3, EPHA3, EPHA7, EPHB3, FGR, GRIN2A, GRIN2B, GRIN2C, LRP1, MAPT, NLGN1, NLGN4X, **Ntrk2,** PIK3C2G, PRKACB, RELN, SHC3, SNCG, STXBP6, SYN2, SYT11, SYT12, SYT8, THBS4, TIAM1
Axonal Guidance Signaling	6.92	ADAM22, ADAM23, ADAM33, ADAMTS15, ADAMTS19, ADAMTS2, ARHGEF6, BMP2, BMP7, CXCL12, DPYSL2, EFNB3, EPHA3, EPHA7, EPHB3, FES, FZD1, GLI1, GNA14, GNAO1, GNG2, ITGA2, ITGA3, ITGA5, L1CAM, LRRC4C, MMP11, MMP14, MMP16, MMP2, MMP28, MMP7, **NGFR,** NRP1, NRP2, NTN4, NTNG1, **Ntrk2, Ntrk3**, PAK6, PGF, PIK3C2G, PLCB4, PLCD3, PLCH2, PLXNB1, PRKACB, PTCH2, SEMA3C, SEMA6A, SLIT3, SMO, TUBA1A, WNT5A
Sperm Motility	6.21	BMX, CALML5, CNGA1, DDR2, EPHA3, EPHB3, FES, FGFR1, FGR, GUCY1A1, KIT, MAP2K6, MERTK, NPPC, NPR1, **Ntrk2, Ntrk3,** PDE2A, PDE4D, PLA2G4A, PLA2G4C, PLAAT3, PLCB4, PLCD3, PLCH2, PRKACB, PTK7, ROR1, SLC12A2, TEC, TEK
Osteoarthritis Pathway	5.75	ACAN, ALPL, BMP2, CCN4, DCN, DDIT4, DDR2, FGF2, FGFR1, FRZB, FZD1, GLI1, H19, IL1B, ITGA2, ITGA3, ITGA5, JAG1, LRP1, NOTCH1, PGF, PRKAG3, PTCH2, PTH1R, RARRES2, SMO, SPHK1, TCF7L1, TIMP3
p53 Signaling	5.56	ADGRB1, BAX, BBC3, CCND1, CCND2, CHEK1, FAS, GADD45A, HDAC9, MDM2, PIK3C2G, SERPINB5, SFN, SNAI2, TNFRSF10A, TP53AIP1, TP63, TRIM29
Breast Cancer Regulation by Stathmin1	5.54	ACKR1, ACKR4, ADGRA2, ADGRB1, ADGRB2, ADGRD2, ADGRG2, ADORA1, ADORA2B, ADORA3, ADRB1, ARHGEF6, BDKRB2, CAMK2B, CCND1, CCND2, CCR10, CMKLR1, CNR1, F2R, FGF2, FZD1, GNG2, GNRHR, GPR146, GPR153, GPR156, GPR173, GPR27, GPR34, GPR61, GPR87, GPRC5D, GPRC6A, HCAR2, HCRTR1, HRH1, HTR4, LGR5, MCHR1, MMP2, NMUR1, NPY1R, OXGR1, OXTR, PGF, PIK3C2G, PLCB4, PPP1R14C, PRKACB, PTH1R, RXFP4, SMO, SSTR2, TAS1R3, TSHR, TUBA1A, VN1R1
Inhibition of Matrix Metalloproteases	5.5	A2M, LRP1, MMP11, MMP14, MMP16, MMP2, MMP28, MMP7, SDC1, TIMP2, TIMP3
cAMP-mediated signaling	5.08	ADCY3, ADCY5, ADORA1, ADORA2B, ADORA3, ADRB1, AKAP12, AKAP3, CALML5, CAMK2B, CNGA1, CNR1, DUSP4, DUSP9, GDPD1, GNAO1, HCAR2, HTR4, NPR3, NPY1R, PDE2A, PDE3A, PDE4D, PKIB, PRKACB, PTH1R, RAP1GAP, RAPGEF4, RXFP4
Role of Osteoblasts, Osteoclasts and Chondrocytes in Rheumatoid Arthritis	4.93	ALPL, BMP2, BMP7, CALML5, CTSK, DKK2, DKK3, FRZB, FZD1, GSN, IL1B, ITGA2, ITGA3, ITGA5, ITGB3, LRP1, MAP2K6, MAPK10, MMP14, **NGFR,** PIK3C2G, SFRP1, SFRP2, SFRP4, SMO, TCF7L1, WIF1, WNT5A
Endocannabinoid Neuronal Synapse Pathway	4.45	ADCY3, ADCY5, CACNA2D1, CACNA2D3, CACNB1, CACNB2, CNR1, GNA14, GNAO1, GNG2, GRIN2A, GRIN2B, GRIN2C, KCNJ5, MAPK10, PLCB4, PLCD3, PLCH2, PRKACB
Adrenomedullin signaling pathway	4.45	ADCY3, ADCY5, ADM, BAX, CALML5, CFH, GNA14, GUCY1A1, IL1B, KCNH2, MAP2K6, MAPK10, MMP2, NPR1, NPR3, PIK3C2G, PLCB4, PLCD3, PLCH2, PRKACB, RAMP3, RXRG, SHC3, TFAP2C, TTN
Coagulation System	4.25	A2M, BDKRB2, F2R, F3, F5, PLAT, SERPINA5, TFPI, VWF
Complement System	4.04	C1QA, C1QC, C1R, C1S, C7, CFB, CFH, CFI, MASP1

Supplementary Table 2. Top 15 dysregulated pathways in IR-SMG

Ingenuity Canonical Pathways	-log(p-value)	Genes
Hepatic Fibrosis / Hepatic Stellate Cell Activation	11.4	A2M, AGT, CCR5, COL16A1, COL17A1, COL18A1, COL20A1, COL23A1, COL4A5, COL4A6, COL5A2, COL6A3, COL7A1, COL8A1, COL9A2, CXCL9, EDNRA, FGF1, FGFR1, FN1, IGF2, IGFBP4, IL1RAPL1, IL1RL1, LAMA1, LBP, LEPR, MMP2, MMP9, **NGFR,** PDGFRB, PGF, TGFB2, TGFB3, TIMP2
Osteoarthritis Pathway	6.44	ACAN, ALPL, CCN4, DCN, DLX5, FGFR1, FGFR3, FN1, FRZB, FZD1, GLI2, GLI3, H19, HTRA1, IL1RAPL1, IL1RL1, ITGA2, ITGA3, JAG1, MMP12, MMP9, NOTCH1, PGF, PTH1R, SMAD6, SMAD9, SOX9, SPP1, TCF7L1
Granulocyte Adhesion and Diapedesis	6.26	CCL18, CCL20, CCL22, CCL28, CLDN1, CLDN10, CLDN11, CX3CL1, CXCL12, CXCL14, CXCL9, IL1RAPL1, IL1RL1, ITGA2, ITGA3, ITGB3, MMP11, MMP12, MMP16, MMP2, MMP7, MMP9, **NGFR,** SDC1, SELP, THY1
Axonal Guidance Signaling	6.08	ADAM22, ADAM23, ADAM33, ADAMDEC1, ADAMTS15, ADAMTS3, ADAMTS8, ARHGEF15, BMP7, CXCL12, EFNB3, EPHA5, EPHB3, FZD1, GLI2, GLI3, GNA14, GNAO1, ITGA2, ITGA3, KCNJ12, L1CAM, MMP11, MMP12, MMP16, MMP2, MMP7, MMP9, NFATC4, **NGF, NGFR,** NRP1, **Ntrk1, Ntrk2,** PAK5, PAPPA, PAPPA2, PGF, PLCD3, PLCD4, PLCH2, PLXNB1, PRKACB, SEMA3B, SEMA3D, SEMA3E, SEMA6D, UNC5B, WNT7B
Sperm Motility	4.98	BMX, CACNA1G, EPHA5, EPHB3, FGFR1, FGFR3, GUCY1A1, KIT, MST1R, **Ntrk1, Ntrk2**, PDE1B, PDE4C, PDGFRB, PLA2G2A, PLA2G2D, PLA2G3, PLA2G4F, PLCD3, PLCD4, PLCH2, PNPLA3, PRKACB, PTK7, ROR1, ROR2, SLC12A2
Inhibition of Matrix Metalloproteases	4.94	A2M, MMP11, MMP12, MMP16, MMP2, MMP7, MMP9, SDC1, THBS2, TIMP2
GP6 Signaling Pathway	4.81	COL16A1, COL17A1, COL18A1, COL20A1, COL23A1, COL4A5, COL4A6, COL5A2, COL6A3, COL7A1, COL8A1, COL9A2, ITGB3, LAMA1, LAMA2, LAMB3, LAMC2, LAMC3
Wnt/β-catenin Signaling	4.5	ACVR1C, AXIN2, CDH1, CDH3, DKK2, DKK3, FRZB, FZD1, GNAO1, MMP7, PPP2R2B, SFRP1, SFRP2, SFRP4, SFRP5, SOX11, SOX6, SOX9, TCF7L1, TGFB2, TGFB3, WNT7B
Synaptogenesis Signaling Pathway	4.3	CACNB1, CAMK2B, CDH1, CDH11, CDH13, CDH15, CDH19, CDH24, CDH3, CDH6, CDH8, COMP, DLG4, EFNB3, EPHA5, EPHB3, GRIN2A, GRIN2C, NECTIN1, NLGN1, NLGN2, NLGN4X, NRXN1, NRXN2, **Ntrk2,** PRKACB, SNAP25, SYT14, SYT7, SYT8, THBS2, THBS4
Agranulocyte Adhesion and Diapedesis	3.79	AOC3, CCL18, CCL20, CCL22, CCL28, CLDN1, CLDN10, CLDN11, CX3CL1, CXCL12, CXCL14, CXCL9, FN1, ITGA2, ITGA3, MMP11, MMP12, MMP16, MMP2, MMP7, MMP9, SELP
Regulation of the Epithelial-Mesenchymal Transition Pathway	3.4	CDH1, FGF1, FGF10, FGF7, FGFR1, FGFR3, FZD1, JAG1, JAG2, LOX, MMP2, MMP9, NOTCH1, PDGFRB, SNAI1, SNAI2, TCF7L1, TGFB2, TGFB3, TWIST2, WNT7B
Role of Osteoblasts, Osteoclasts and Chondrocytes in Rheumatoid Arthritis	3.38	ALPL, BMP7, DKK2, DKK3, DLX5, FRZB, FZD1, IL1RAPL1, IL1RL1, ITGA2, ITGA3, ITGB3, NFATC4, **NGFR,** SFRP1, SFRP2, SFRP4, SFRP5, SMAD6, SMAD9, SPP1, TCF7L1, WNT7B
Breast Cancer Regulation by Stathmin1	3.26	ADGRA1, ADGRA2, ADGRB1, ADGRB2, ADGRD2, ADGRE1, ADGRF1, ADGRL3, ADORA1, ADORA2B, ADRB1, ARHGEF15, ARHGEF4, CAMK2B, CCR5, CDK1, CX3CR1, E2F7, EDNRA, F2RL2, FZD1, GLP1R, GPR153, GPR173, GPR26, GPR27, GPR37L1, GPR83, GPR87, GPRC6A, LGR5, MCHR1, MMP2, MMP9, OXGR1, OXTR, P2RY2, PGF, PPP1R14C, PPP2R2B, PRKACB, PTGIR, PTH1R, SLC52A1, SSTR2, TAS1R3, TGFB2
Bladder Cancer Signaling	3.12	CDH1, CDKN1A, FGF1, FGF10, FGF7, FGFR3, MMP11, MMP12, MMP16, MMP2, MMP7, MMP9, PGF
Transcriptional Regulatory Network in Embryonic Stem Cells	2.99	FOXD3, H4C13, H4C2, H4C3, H4C4, H4C6, H4C8, L1CAM, TCF7L1

Supplementary Table 3. Common predicted upstream regulators and their downstream target molecules

Upstream Regulator	LogFC (PG)	LogFC (SMG)	Molecule Type	Predicted activation state	p-value (PG)	p-value (SMG)	Target Molecules in PG dataset	Target Molecules in SMG dataset
SFRP2	2.81	3.485	transmembrane receptor		2.57E-02	2.20E-02	ADORA1, ALDH1A2, BMP2, BMP7, CAV2, COL5A2, F3, GJA1, IGF2, IGFBP4, IGFBP6, IL1B, ITGA5, JAG1, LTF, MMP2, POMC, WNT5A	MMP2, SNAI2
**NGFR**	2.794	1.855	transmembrane receptor		5.39E-03	9.79E-05	ALDH1A2, APOD, ASPA, BMP7, CRABP2, CX3CL1, CXCL12, FABP7, FGF2, GFAP, GPNMB, GSN, HEYL, IFIT3, IL1B, LAMC1, MFGE8, MICOS10-NBL1/NBL1, MMP2, OLFM4, PLP1, POSTN, RELN, SDK2, SELP, SERPINF1, SOX4, SPHK1, SPON2, TFAP2C	A2M, CCNA2, IL1RL1, NCAM1, NGF, NGFR, PLAT, SNAP25
**Ntrk2**	2.522	1.953	kinase	Activated	1.84E-02	3.12E-02	BAX, CAV1, ETV4, KRT5, MMP14, MMP7	CCNA2, CDH1, CDK1, CDKN1A, HEY2, JAG1, SNAP25, SPP1
ETV5	2.263	1.618	transcription regulator		2.37E-07	2.64E-05	BAX, CAV1, CAVIN1, CAVIN2, CCND1, CXCL12, DPYSL2, ETV5, ITGA5, KRT19, LCN2, MMP14, MMP2, PMP22, S100A14, S100A6, SAMHD1, SNAI2, SOAT1, TIMP3	AGRN, CDH1, CLDN1, CXCL12, ETV5, FN1, KRT19, LCN2, MMP2, NCAM2, NRCAM, PMP22, S100A14, SNAI2, SOAT1, SPP1
BMP7	2.18	1.953	growth factor		5.69E-03	1.23E-07	APOE, CCN4, CCN5, CCND1, DIO3, FGFR1, FZD1, GCLM, GSTM5, IL1B, NOTCH1, PMEPA1, POSTN, SNAI2, SOX2, SSTR2, TGM2, TIMP3	ADORA1, ALDH1A2, BIRC5, BMP7, CDH1, CDKN1A, COL5A2, CYP19A1, DLX5, DSP, EPCAM, FN1, IGF2, IGFBP4, JAG1, LTF, MMP2, MMP9, MST1R, NCAM1, PDGFRB, PRDM16, SMAD6, SOSTDC1, SPP1, TGFB3, VCAN
PGF	1.907	1.272	growth factor		5.61E-03	1.41E-04	CTSB, LFNG, MMP14, S100A6, SOX2, TIMP3, TSPAN8	BIRC5, CCL20, CCL28, CDK1, DCN, DNM3OS, KIT, MMP9, NANOS1
ITGB3	1.865	1.404	transmembrane receptor		1.31E-02	8.01E-03	BAX, BMP7, CCND1, CCND2, EIF3C, FGF1, FGF10, FGF2, FGF7, FGFR1, MMP14, NOTCH1, PLP1, SFN, SPARC	CDK1, FN1, ITGA3, ITGB3, MMP2, MMP9, SELP, SNAI2, SPARC
TP63	1.815	1.998	transcription regulator	Activated	1.06E-17	3.04E-11	BMP2, CCND1, CTSV, FABP7, GADD45A, H19, IGF2, IGFBP2, IGFBP4, IGFBP6, ITGA5, MDM2, MMP11, OGN, PHLDA1, RAD51, ZFAND5	AGR2, BMP7, CA4, CCNA2, CCNB1, CDH1, CDH3, CDK1, CDKN1A, DKK3, ECM1, FGFR3, FN1, GPX2, GRHL2, HAS3, HTRA1, IGFBP2, IGFBP7, ITGA2, ITGA3, ITGA7, JAG1, JAG2, KRT6B, L1CAM, MFGE8, MIR155HG, MIR205HG, MPZL2, NALCN, NOTCH1, NT5E, P2RY2, PAX9, PI3, PKP1, POSTN, RGS13, RIN1, SERPINB2, SERPINB5, SERPINF1, SNAI1, SNAI2, SOX9, SPON1, ST8SIA6, SYT7, TGFB2, TGFB3, THBS2, THY1, TNFSF15, TP53AIP1, TP63, TP73, TRIM29, VDR, WFDC2
ETV4	1.811	1.101	transcription regulator		1.14E-02	3.02E-04	IL1B, ITGA3, ITGA5, ITGB3, MMP14, MMP2, SELP, SNAI2, SPARC	CDH1, ETV4, FN1, KRT5, MMP7, MMP9, MUC4, SPP1
TFAP2C	1.711	2.163	transcription regulator		4.67E-02	3.69E-03	CCND1, CCND2, HEYL, ITGB3, SNAI2, SOX2, TIMP3	CDH1, CDKN1A, EGR3, JAG1, KRT81, MMP2, MMP9, NRP1, PITX2, SEMA3B
SNAI2	1.488	1.298	transcription regulator		6.76E-03	1.09E-09	BMP2, BMP7, CCL20, IL1B, JAG1, NOTCH1	CDH1, CDKN1A, CLDN1, CXCL12, DSP, EGR2, EPCAM, FN1, ID4, ITGA3, L1CAM, MMP9, NES, SNAI2, SOX9, TP63, TWIST2, VDR
IGF2	1.458	1.36	growth factor		9.80E-03	3.44E-05	A2M, CCND1, GFAP, GRIN2B, NGFR, PLAT	BIRC5, CDK1, CDKN1A, CORO2B, CTSV, CYP1B1, EGR2, FABP7, FN1, H19, IGF2, IGFBP2, IGFBP4, MMP11, MMP12, MYBL2, NGF, OGN, PRDM16, SEZ6L2, SPP1, THBS2

CX3CL1	1.414	1.374	cytokine	Activated	1.11E-11	1.22E-07	CAV1, COL18A1, FGF1, ITGA5, ITGB3, KIT, MMP2, NPR1, PGF, SELP, TEK, TFPI	ALDH1A2, APOD, ASPA, BMP7, CRABP2, CX3CL1, CXCL12, FABP7, HEY2, MFGE8, MMP2, MMP9, OLFM4, PLP1, POSTN, PROM1, SDK2, SELP, SERPINF1, SPP1, TFAP2C, TGFB2, TGFB3
NOTCH1	1.348	1.21	transcription regulator		5.41E-04	3.50E-03	AKR1C1/AKR1C2, BMP2, CCND1, CCND2, FABP7, FGF2, GATD3A/GATD3B, GFAP, GLI1, HBA1/HBA2, HEYL, HSPH1, IGFBP2, IGFBP4, JAG1, LFNG, LOX, MCAM, MMP2, NGFR, NOTCH1, NRG1, PTGDS, RND3, TEK, TFF3, TP63, WNT5A	CDH1, CDKN1A, ESM1, FABP7, GATA2, GATD3A/GATD3B, GLI2, HEY2, IGFBP2, IGFBP4, JAG1, LOX, MCAM, MMP2, NGFR, NOTCH1, PDGFRB, SLC5A5, SNAI1, SPP1, TFF3, TGFB2, TGFB3, TP63
FGF10	1.33	1.151	growth factor		2.66E-02	5.31E-03	ABCB1, ABCG2, BMP2, CCND1, CCND2, FGF2, JAG1, POMC, PYCARD	ANXA1, CYP19A1, CYP1B1, MMP9, SNAI1, SOX9, TACSTD2, TSPAN8
FGF1	1.317	1.16	growth factor		1.31E-04	3.61E-06	CCL20, CCL28, DCN, IL1B, KIT, NANOS1, NRP2	ADGRE1, AXIN2, CCN4, CCN5, CDH1, CDH11, CDKN1A, CPXM1, DIO3, FGFR1, FZD1, MAB21L1, NOTCH1, POSTN, SLC7A2, SNAI2, SPP1, SSTR2, TGFB2, TGFB3
FGFR1	1.191	1.056	kinase	Activated	1.71E-04	8.62E-04	MMP2, SNAI2	BIRC5, BMP7, CDKN1A, DCX, DDX25, FGF1, FGF10, FGF7, FGFR1, FGFR3, NOTCH1, PLP1, SPARC
JAG2	1.156	1.218	growth factor	Activated	1.66E-02	2.69E-03	BBC3, CCND1, CXCL12, ITGA3, L1CAM, MMP14, NES, SNAI2, TP63	BMP7, CCL20, CXCL9, JAG1, MMP12, NOTCH1, SPP1
JAG1	1.103	1.097	growth factor	Activated	2.43E-02	9.85E-06	F2R, FOXA1, JAG1, MMP14, MMP2, MT1F, NRP1, TACC1	CDKN1A, CYP19A1, GATA2, GLI2, HEY2, ITGB3, MMP9, PDGFRB, SNAI1, SNAI2, SPP1, TGFB3
NOSTRIN	-1.399	-1.074	transcription regulator	Inhibited	4.33E-08	1.68E-05	ABCB1, ADM, AEN, AGR2, ALDH1A3, ALOX12, BAX, BBC3, BDKRB2, BLM, BMP7, BST2, CAVIN2, CCND1, CCND2, CDH3, COL4A1, CYGB, DDIT4, DKK3, DOK1, F2R, F3, FAS, GADD45A, GPNMB, IGFBP2, IGFBP6, IGFBP7, IL1B, INHBA, ITGA2, ITGA3, ITGA7, JAG1, JAG2, KCNG1, KHK, KRT6B, L1CAM, MDM2, MFGE8, MIR205HG, MMP14, NALCN, NOTCH1, PARD6G, PI3, PKP1, POSTN, PRNP, PTPN13, RAD51, RBBP8, RBMS3, RELN, RGS13, RIN1, S100A2, SERPINB2, SERPINB5, SERPINF1, SFN, SNAI2, SOX2, SOX4, SPON1, SSPN, STON2, TIMP3, TNFRSF10A, TNFSF15, TP53AIP1, TP63, TRIM29, WNT5A	COL18A1, FGF1, FN1, ITGB3, KIT, MMP2, MMP9, PGF, SELP

Supplementary Table 4. Antibodies

Antibodies			
Anti-calponin	1:100	Abcam	Ab46794
Anti-E-cadherin (2AE10)	1:100	Cell Signaling	mAb#3195
Anti-Smooth muscle actin	1:200	Millipore Sigma	A2547
Anti-Smooth muscle actin-Cy3 conjugate	1:100	Millipore Sigma	C6198
Anti-Cytokeratin 5	1:500	Covance	PRB-160P
Cytokeratin-14	1:200	Covance	PRB-155P
Troma-III, Keratin 19 antibody	1:100	DSHB	Troma-III-c conc
Anti-NGF	1:200	Alomone Labs	AN-240
Anti-Ngfr	1:100	LSBio	B531
Anti-Tubb3	1:200	R&D	MAB1195
Anti-TrkA	1:100	MyBiosource	MBS5400171
Anti-TrkB	1:100	Cell signaling	80G2
Anti-Trk-C	1:100	Cell signaling	C44H5
Anti-Mist1	1:250	Abcam	ab187978
Anti-Nkcc1	1:250	Santa Cruz Biotechnology	sc-21545
Anti-Cnn1	1:250	LSBio	B4304
Anti-CollIV	1:100	EMD Millipore	AB756P
Anti-FGFR1	1:100	GeneTex	GTX10646
Anti-FGFR1	1:100	Cell signaling	9470S
Alexa Fluor® 488 AffiniPure F(ab')$_2$ Fragment Donkey Anti-Goat IgG (H+L)	1:250	Jackson Immunoresearch Laboratories	705-546-147
Alexa Fluor® 647 AffiniPure F(ab')$_2$ Fragment Donkey Anti-Goat IgG (H+L)	1:250	Jackson Immunoresearch Laboratories	705-606-147
Alexa Fluor® 488 AffiniPure F(ab')$_2$ Fragment Donkey Anti-Rabbit IgG (H+L)	1:250	Jackson Immunoresearch Laboratories	711-546-152
Cy™3 AffiniPure F(ab')$_2$ Fragment Donkey Anti-Rabbit IgG (H+L)	1:250	Jackson Immunoresearch Laboratories	711-166-152
Cy™3 AffiniPure F(ab')$_2$ Fragment Donkey Anti-Mouse IgG (H+L)	1:250	Jackson Immunoresearch Laboratories	715-165-150
Hoechst	1:1000	Thermo Fisher Scientific	H3570
DAPI (Dihydrochloride)	1:10000	Millipore Sigma	268298

Supplementary Table 5. Oligonucleotides and probes

**Human primers**			
NTRK1	CCTGACACTAACAGCACATCTGGAGAC	Life Technologies	N/A
	TGAGCACAAGGAGCAGCGTAGAA		
NTRK2	TGGCTGGTTGTGGGCTTCTG	Life Technologies	N/A
	AATGCCACGATGCCAGGAGAA		
NTRK3	GAAGCGTCTGGCTGGACTATGTG	Life Technologies	N/A
	GTGGTGAGCCTGGTGGAGCCTGAG		
NGFR	GACTCTACACTGTGAACTTG	Life Technologies	N/A
	CTCCGATCCTCTTCATCTTA		
NGF	CAACAGGACTCACAGGAGCA	Life Technologies	N/A
	GTCTGTGGCGGTGGTCTTAC		
BMP7	CCTTGCCTTGGCTGGTGAGTC	Life Technologies	N/A
	CAGGAACTGTCTGAGTCAAGATGGAGAA		
FGFR1B	CCGACAAAGAGATGGAGGTGCTTCA	Life Technologies	N/A
	CCAGAACGGTCAACCATGCAGAGT		
FGFR1	TTGAGACCGCACAGGAGT	Life Technologies	N/A
	GAGTAGTAACAGCAAGGACATCAC		
JAG1	GCTCTGTCTTAACTGTGGCTTGGAT	Life Technologies	N/A
	CGTTGTTGGTGGTGTTGTCCTCAG		
WNT5A	CACAGGTTCTCAGCCCAAGCAACAAGGT	Life Technologies	N/A
	GCCAGCATCACATCACAACACGGAGGAATC		
PTCH2	CAGGAGGAGGAGACAAGGCAGAAGG	Life Technologies	N/A
	GGGCAAACTGATAGCGGGCGAAT		
GAPDH	CAGCCTCAAGATCATCAGCA	Life Technologies	N/A
	TGTGGTCATGAGTCCTTCCA		
**Mouse primers**			
Acta2	GCATGGATGGCATCAATCAC	Life Technologies	N/A
	ACCTATCTGGTCACCTGTATGTA		
Cnn1	CGCACAACTACTACAACTC	Life Technologies	N/A
	CCCAAACCGTAACCCTATA		
Ngf	TTGATCGGCGTACAGGCAGAACC	Life Technologies	N/A
	CGGAGGGCTGTGTCAAGGGAATG		
Ngfr	TCCACACTCCTTCTCTTACACATA	Life Technologies	N/A
	TTGACGCCCTCATTCAGAAAG		
Ntrk1	TGTCCAAGTCAGCGTCTCCTT	Life Technologies	N/A
	GTTGAGCACAGAGCCGTTGAA		
Ntrk2	GACACGCACTCCGACTGACT	Life Technologies	N/A
	CCAAGACCAGCAGGCATAAGC		
Ntrk3	GTGGTGAGCCTGGTGGAGCCTGAG	Life Technologies	N/A
	AGTGGAGCGTGGGTGTCGGGTTG		
Ntf3	CGGCAACAGAGACGCTACAATTCG	Life Technologies	N/A

	TCCGTGGTGAGGTTCTATTGGCTAC		
Ntf5	GAGGAGGTGGAGGTGCTGCTGTT	Life Technologies	N/A
	GGGACGCTGGGGAGGAGGAA		
Fgfr1b	AGAGCGGGGAGTATGTGTGTAAGGTTTC	Life Technologies	N/A
	TGGTGACAGTGAGCCACGCAGAC		
Fgfr2b	TGGCTCTGTTCAATGTGACGGAGATGGATG	Life Technologies	N/A
	AGGCGCTTGCTGTTTGGGCAGGAC		
Aqp5	TCTACTTCTACTTGCTTTTCCCCTCCTC	Life Technologies	N/A
	CGATGGTCTTCTTCCGCTCCTCTC		
Bhlha15	TCGCTGACCGCCACCATACTTAC	Life Technologies	N/A
	CTGCTGCTGCTGCTGCTGTTG		
Prol1	ACC ACA CCA GCA ACA ACC ACA A	Life Technologies	N/A
	TGG CTG TAG AGG TGC TAG GCT TAG		
Bpifa2	GCTGTCTTCCAACGGCAATGGCATT	Life Technologies	N/A
	CCAATGAAAGGCAGAACCAAGGAGGCTTC		
Smgc	TCCTCAGCAGTAGACTCCACAGATTC	Life Technologies	N/A
	TGAAAGATCCACCATTATTCTCTCCAACC		
Krt19	GCCACCTACCTTGCTCGGATTG	Life Technologies	N/A
	GTCTCTGCCAGCGTGCCTTC		
Krt14	CCTCATCCTCTCAATTCTCCTCTGGCTCTC	Life Technologies	N/A
	CTTGGTGCGGATCTGGCGGTTGG		
Krt5	TCCTGTTGAACGCCGCTGAC	Life Technologies	N/A
	CGGAAGGACACACTGGACTGG		
Il17b	CAGGAGGACCGTAGCATGGT	Life Technologies	N/A
	TTGGTGGTTGGCTCAGAAGATG		
Sox10	GCTGGACCGCACACCTTGG	Life Technologies	N/A
	GCCGCCGCCGCTTCG		
Mki67	TTGCCTCCTAATACACCACTGA	Life Technologies	N/A
	CCGTTCCTTGATGATTGTCTTGA		
Rs29	GGAGTCACCCACGGAAGTTCGG	Life Technologies	N/A
	GGAAGCACTGGCGGCACATG		
RNA probe for Cnn1		ACDBio	N/A
RNA probe for Ntrk1		ACDBio	N/A
RNA probe for Ntrk2		ACDBio	N/A
RNA probe for Ntrk3		ACDBio	N/A
RNA probe for Ngfr		ACDBio	N/A

Supplementary Table 6. Chemicals, Peptides, and Recombinant Proteins

DMEM-F12	1x	Thermo Fisher Scientific	11320-082
Penicillin-streptomycin	1x	Thermo Fisher Scientific	15140122
Recombinant human FGF10 protein	100 ng/mL	R &D Systems Inc.	345-FG-025
Recombinant human EGF protein	20 ng/mL	R &D Systems Inc	236-EG-200
Recombinant human FGF basic/FGF2 protein	20 ng/mL	R &D Systems Inc	233-FB-025
Recombinant human SCF protein	100 ng/mL	R &D Systems Inc	455-MC
Recombinant mouse beta NGF protein	1-100 ng/mL	R &D Systems Inc	1156-NG-100
Smooth muscle cell growth media	1X	Cell Applications, Inc.	311-200
Smooth muscle cell differentiation media	1X	Cell Applications, Inc.	311D-250
N2 supplement	1X	Thermo Fisher Scientific	17502001
Dexamethasone	1 mM	Sigma Aldrich Corp	D2915
Y27632- rock inhibitor	5 mM	Sigma Aldrich Corp	Y0503
Hyaluronidase from bovine testes	1 mg/mL	Sigma Aldrich Corp	H3506
Carbachol (Cch)	10 nM	Sigma Aldrich Corp	C4382
Collagenase Type II	0.575 mg/ml	Thermo Fiscer Scientific	17101015
ITS-G	1X	Thermo Fisher Scientific	41400045
Calcium chloride	6.25 mM	Quality Biological	351-130-721
TrypLE™ Express Enzyme (1X), phenol red	1X	Thermo Fisher Scientific	12605036
Vitamin C	150 µg/mL	Thermo Fisher Scientific	11107047
Transferrin	50 µg/mL	Sigma Aldrich Corp	A4544
GNF5837		R &D Systems Inc	4559/10
RO2750		R &D Systems Inc	2272/1
Dimethyl sulfoxide (DMSO)		Millipore Sigma	34869-100ML
Fluoro-Gel with TRIS mounting media		Thermo Fisher Scientific	50-247-04
Paraformaldehyde (formaldehyde) Aqueous solution		Electron Microscopy Sciences	15710
Xylene substitute		Millipore Sigma	A5597-1GAL
Ethanol 100%		Millipore Sigma	E7023
Ethanol 95%		Millipore Sigma	493538
Acetone		Millipore Sigma	179973
Methanol		Fisher Scientific	A412-4
**Critical Commercial Assays**			
M.O.M (Mouse on Mouse) Immunodetection Kit		Vector Laboratories	MKB-2213-1

RNAqueous-4PCR kit and DNase removal reagent	Thermo Fisher Scientific	AM1914
RNAqueous Micro Kit	Thermo Fisher Scientific	AM1931
Superscript III First-Strand Synthesis System	Life Technologies	18080051
iQ SYBR Green Supermix	Bio-Rad	1708882
MACS SMART Strainers 70µm	Miltenyi Biotech	130-110-916
24 well BioCoat^{TM} Collagen IV multiwell plates	Corning	08-774-29
8 well collagen IV coated 15-m slide	Ibidi	50-305-885
15ml gentleMACS C tube	Miltenyi Biotech	130-093-237
Corning® Costar® Ultra-Low Attachment Multiple Well Plate	Millipore Sigma	CLS3473
Whatman® Nuclepore™ Track-Etched Membranes	Millipore Sigma	WHA110405
50 mm Dish, No. 1.5 Coverslip, 14 mm Glass Diameter, Uncoated	MatTek	P50G-1.5-14-F

Supplementary Table 7. Software

Beacon Designer	PREMIER Biosoft	N/A
R & R studio	https://rstudio.com/	N/A
FIJI	Schindelin et al. 2012	N/A
Photoshop	Adobe	N/A
Excel	Microsoft	N/A
Word	Microsoft	N/A
Powerpoint	Microsoft	N/A
NDP.view2	Hakamatsu	U12388-01
