# Supplementary material for: Neurotrophin signaling is a central mechanism of salivary dysfunction after irradiation that disrupts myoepithelial cells
Source: NPJ Regen Med. 2023 Mar 25;8:17. doi: 10.1038/s41536-023-00290-7 (PMC10039923; doi:10.1038/s41536-023-00290-7)
Supplement: Supplementary file 5 — Reporting Summary [file 41536_2023_290_MOESM5_ESM.pdf]

nature portfolio

Corresponding author(s): Alejandro Martinez Chibly

Last updated by author(s): Jan 5, 2023

Reporting Summary

Nature Portfolio wishes to improve the reproducibility of the work that we publish. This form provides structure for consistency and transparency in reporting. For further information on Nature Portfolio policies, see our Editorial Policies and the Editorial Policy Checklist.

Statistics

For all statistical analyses, confirm that the following items are present in the figure legend, table legend, main text, or Methods section.

n/a	Confirmed	
☐	☒	The exact sample size (*n*) for each experimental group/condition, given as a discrete number and unit of measurement
☐	☒	A statement on whether measurements were taken from distinct samples or whether the same sample was measured repeatedly
☐	☒	The statistical test(s) used AND whether they are one- or two-sided<br>*Only common tests should be described solely by name; describe more complex techniques in the Methods section.*
☐	☒	A description of all covariates tested
☐	☒	A description of any assumptions or corrections, such as tests of normality and adjustment for multiple comparisons
☐	☒	A full description of the statistical parameters including central tendency (e.g. means) or other basic estimates (e.g. regression coefficient) AND variation (e.g. standard deviation) or associated estimates of uncertainty (e.g. confidence intervals)
☐	☒	For null hypothesis testing, the test statistic (e.g. *F*, *t*, *r*) with confidence intervals, effect sizes, degrees of freedom and *P* value noted<br>*Give P values as exact values whenever suitable.*
☒	☐	For Bayesian analysis, information on the choice of priors and Markov chain Monte Carlo settings
☒	☐	For hierarchical and complex designs, identification of the appropriate level for tests and full reporting of outcomes
☒	☐	Estimates of effect sizes (e.g. Cohen's *d*, Pearson's *r*), indicating how they were calculated

Our web collection on statistics for biologists contains articles on many of the points above.

Software and code

Policy information about availability of computer code

Data collection

Data analysis

A complete list of the packages used for analysis is provided in this github repo: https://github.com/chiblya/IR_SalivaryGlands
Rstudio 1.4 was used for analysis, along with the following packages:
[1] corrplot_0.92 fgsea_1.20.0 gage_2.44.0
[4] viridis_0.6.2 viridisLite_0.4.0 pheatmap_1.0.12
[7] reshape2_1.4.4 superheat_0.1.0 Matrix_1.3-4
[10] Hmisc_4.6-0 Formula_1.2-4 survival_3.3-1
[13] lattice_0.20-44 RUVSeq_1.28.0 EDASeq_2.28.0
[16] ShortRead_1.52.0 GenomicAlignments_1.30.0 Rsamtools_2.10.0
[19] Biostrings_2.62.0 XVector_0.34.0 BiocParallel_1.28.3
[22] gplots_3.1.1 DESeq2_1.34.0 RColorBrewer_1.1-2
[25] edgeR_3.36.0 limma_3.50.1 SeuratObject_4.0.4
[28] Seurat_4.1.0 ggpubr_0.4.0 tidyr_1.2.0
[31] ggplot2_3.3.5 dplyr_1.0.8 SummarizedExperiment_1.24.0
[34] Biobase_2.54.0 GenomicRanges_1.46.1 GenomeInfoDb_1.30.1
[37] IRanges_2.28.0 S4Vectors_0.32.4 BiocGenerics_0.40.0
[40] MatrixGenerics_1.6.0 matrixStats_0.61.0

For manuscripts utilizing custom algorithms or software that are central to the research but not yet described in published literature, software must be made available to editors and reviewers. We strongly encourage code deposition in a community repository (e.g. GitHub). See the Nature Portfolio guidelines for submitting code & software for further information.

Data

Policy information about availability of data

All manuscripts must include a data availability statement. This statement should provide the following information, where applicable:

- Accession codes, unique identifiers, or web links for publicly available datasets
- A description of any restrictions on data availability
- For clinical datasets or third party data, please ensure that the statement adheres to our policy

RNAseq data are deposited to GeneExpression Omnibus (GSE206878). All code related to the analysis has been deposited to a publicly available repository located in https://github.com/chiblya/IR_SalivaryGlands

Human research participants

Policy information about studies involving human research participants and Sex and Gender in Research.

Reporting on sex and gender	Biopsies from control and irradiated PG and SMG were collected from volunteers undergoing surgery for head and neck cancer at the Princess Margaret Hospital (Toronto, CA). Both sexes were included in the study although higher number of male-derived samples were analyzed due to the higher prevalence of head and neck cancer in males. PCA analysis did not reveal sex-dependent clustering, and thus salivary glands from both sexes were combined for analysis.
Population characteristics	The recorded patient characteristics are described in Supplementary Figure 1B.
Recruitment	Samples were collected from volunteers undergoing surgery for head and neck cancer at the Princess Margaret Hospital (Toronto, CA)
Ethics oversight	The Institutional Review Boards approved all research procedures and the study participants gave written informed consent (Protocol #UHN 2016-0486 & REB #11-0988-CE).

Note that full information on the approval of the study protocol must also be provided in the manuscript.

Field-specific reporting

Please select the one below that is the best fit for your research. If you are not sure, read the appropriate sections before making your selection.

☒ Life sciences ☐ Behavioural & social sciences ☐ Ecological, evolutionary & environmental sciences

For a reference copy of the document with all sections, see nature.com/documents/nr-reporting-summary-flat.pdf

Life sciences study design

All studies must disclose on these points even when the disclosure is negative.

Sample size	No sample-size calculation was performed. A minimum n=3 was included for all analyses to perform statistical comparisons, but usually more samples were evaluated. For organ cultures, the numbers are chosen to minimize the animal use burden of the study while maximizing the number of treatments that were performed in a single experiment. Given that embryos are used, the number of glands for a given experiment depends on the number of embryos in a litter, where all embryos from 1 litter are used in a single experiment and multiple litters combined to account for batch effects and biological variation. For MEC cultures, the initial cell preparation is pooled from multiple animals from a single litter to account for biological variation.
Data exclusions	Biopsies with a RIN<7 were excluded from RNAseq analysis. In addition, 4 samples were presumed outliers based on their abnormal library size and PCA analysis, and they were excluded from downstream analysis.
Replication	All experiments were repeated at least twice and all data are reported.
Randomization	For each experiment (both organ cultures and MEC cultures) mice from a single litter were used for a single experiment, and thus all treatments were assigned at random. Sex was not determined given that we used embryos and P2 mice for the study; however, the number of glands and random distribution of sex in a litter ensures that both are represented. For MEC cultures, cells from multiple mice are pooled before plating thus accounting for biological variation between multiple animals.
Blinding	Sequencing and quantification of immunofluorescence images was blinded. However, note that the morphological differences in irradiated tissues are often easily distinguishable and thus complete blinding is not possible.

Reporting for specific materials, systems and methods

We require information from authors about some types of materials, experimental systems and methods used in many studies. Here, indicate whether each material, system or method listed is relevant to your study. If you are not sure if a list item applies to your research, read the appropriate section before selecting a response.

Materials & experimental systems

n/a	Involved in the study	
☐	☒	Antibodies
☒	☐	Eukaryotic cell lines
☒	☐	Palaeontology and archaeology
☐	☒	Animals and other organisms
☐	☒	Clinical data
☒	☐	Dual use research of concern

Methods

n/a	Involved in the study	
☒	☐	ChIP-seq
☒	☐	Flow cytometry
☒	☐	MRI-based neuroimaging

Antibodies

Antibodies used	All antibodies used in the study are provided in the resource table (Supplementary File S4).
Validation	The antibodies for Smooth muscle actin, E-cadherin, cytokeratins 5, 14, and 19, Tubb3, Mist1, NKCC1, FGFR2, CNN1, and ColIV have been widely used and validated in salivary gland research (Nelson et al, 2013; Aure et al, 2015; Lombaert et al, 2013; Lombaert et al 2020). Antibodies for TrkA, TrkB, TrkC, and NGFR were tested against a matching IgG control in both mouse and human tissues to rule out non-specific binding. All antibodies were developed for histology in mouse and human according to the respective vendors.

Animals and other research organisms

Policy information about studies involving animals; ARRIVE guidelines recommended for reporting animal research, and Sex and Gender in Research

Laboratory animals	Timed-pregnant ICR females were purchased from Envigo at gestational day E9. Mouse embryos were collected from ICR pregnant females at embryonic day E16 for SMG organ cultures and immunostaining, and newborn pups were collected at P1, P2, and P8 for immunostaining and isolation and culture of MECs.
Wild animals	The study did not involve wild animals
Reporting on sex	For each experiment (both organ cultures and MEC cultures) mice from a single litter were used for a single experiment. Sex was not determined given that we used embryos and P2 mice for the study; however, the number of glands and random distribution of sex in a litter ensures that both are represented. For MEC cultures, cells from multiple mice are pooled before plating thus accounting for biological variation between multiple animals.
Field-collected samples	Samples were not collected from field
Ethics oversight	All mice were maintained and treated according to guidelines approved by the National Institute of Dental and Craniofacial Research and National Institutes of Health Animal Care and Use Committee (ASP#10-2022)

Note that full information on the approval of the study protocol must also be provided in the manuscript.

Clinical data

Policy information about clinical studies

All manuscripts should comply with the ICMJE guidelines for publication of clinical research and a completed CONSORT checklist must be included with all submissions.

Clinical trial registration	NA
Study protocol	NA
Data collection	NA
Outcomes	NA
